# Defining Discriminatory Antibody Fingerprints in Active and Latent Tuberculosis

**DOI:** 10.3389/fimmu.2022.856906

**Published:** 2022-04-20

**Authors:** Nadege Nziza, Deniz Cizmeci, Leela Davies, Edward B. Irvine, Wonyeong Jung, Brooke A. Fenderson, Marwou de Kock, Willem A. Hanekom, Kees L. M. C. Franken, Cheryl L. Day, Tom H. M. Ottenhoff, Galit Alter

**Affiliations:** ^1^ Ragon Institute of Massachusetts General Hospital (MGH), Massachusetts Institute of Technology (MIT) and Harvard, Cambridge, MA, United States; ^2^ Division of Infectious Diseases, Brigham and Women’s Hospital, Boston, MA, United States; ^3^ Department of Immunology and Infectious Diseases, Harvard T.H. Chan School of Public Health, Boston, MA, United States; ^4^ South African Tuberculosis Vaccine Initiative (SATVI) and School of Child and Adolescent Health, Institute of Infectious Diseases and Molecular Medicine, University of Cape Town, Cape Town, South Africa; ^5^ Africa Health Research Institute, Durban, South Africa; ^6^ Division of Infection and Immunity, University College London, London, United Kingdom; ^7^ Department of Infectious Disease, Leiden University, Leiden, Netherlands; ^8^ Department of Microbiology and Immunology, Emory University School of Medicine, Emory University, Atlanta, GA, United States

**Keywords:** active and latent tuberculosis, antibodies, HIV, biomarkers, diagnostics

## Abstract

Tuberculosis (TB) is among the leading causes of death worldwide from a single infectious agent, second only to COVID-19 in 2020. TB is caused by infection with *Mycobacterium tuberculosis* (Mtb), that results either in a latent or active form of disease, the latter associated with Mtb spread. In the absence of an effective vaccine, epidemiologic modeling suggests that aggressive treatment of individuals with active TB (ATB) may curb spread. Yet, clinical discrimination between latent (LTB) and ATB remains a challenge. While antibodies are widely used to diagnose many infections, the utility of antibody-based tests to diagnose ATB has only regained significant traction recently. Specifically, recent interest in the humoral immune response to TB has pointed to potential differences in both targeted antigens and antibody features that can discriminate latent and active TB. Here we aimed to integrate these observations and broadly profile the humoral immune response across individuals with LTB or ATB, with and without HIV co-infection, to define the most discriminatory humoral properties and diagnose TB disease more easily. Using 209 Mtb antigens, striking differences in antigen-recognition were observed across latently and actively infected individuals that was modulated by HIV serostatus. However, ATB and LTB could be discriminated, irrespective of HIV-status, based on a combination of both antibody levels and Fc receptor-binding characteristics targeting both well characterized (like lipoarabinomannan, 38 kDa or antigen 85) but also novel Mtb antigens (including Rv1792, Rv1528, Rv2435C or Rv1508). These data reveal new Mtb-specific immunologic markers that can improve the classification of ATB versus LTB.

## Introduction

Infection with *Mycobacterium tuberculosis* (Mtb) affects one quarter of the world’s population (WHO report 2020) and causes approximately 1.5 million deaths annually (WHO global TB report). While the majority of infected individuals control the infection for life, in a state referred to as latent tuberculosis infection (LTB) (1, 2), approximately 5-10% of the infected individuals lose control of the bacterial infection. Consequently, these individuals progress to active tuberculosis (ATB) disease, and if untreated contribute centrally to dissemination of the infection and may ultimately succumb to death (3–5). Moreover, in the setting of HIV co-infection, the risk of developing ATB is 20 to 30 times higher, and without treatment the mortality rate of ATB reaches nearly 100% (3). Thus, diagnostic tools are urgently needed to help guide clinical care across populations.

Current diagnostic tests include assays of bacteriological presence (microscopic or genetic), supplemented by the analysis of symptoms, radiological evidence and tests that involve the detection of recall-memory to Mtb antigens based on the tuberculin skin test (TST) or the ex vivo detection of memory Mtb-specific T-cells based on the interferon-γ (IFN-γ) release assay (IGRA) (6, 7). While TST and IGRA clearly capture Mtb exposure, current diagnostics distinguish ATB and LTB poorly, rendering clinical management of TB disease a major challenge in curbing deaths and disease spread. Thus, improved TB diagnostics are urgently needed to reduce morbidity and mortality.

Pathogen-specific antibodies represent critical markers of infection and disease across many infectious disorders (8–10). Since 1983, antibody responses have been studied for the serodiagnosis of TB (11–16). In addition, studies on HLA-linked immune response highlighted association between sputum smear-positive TB and expression of Class II HLA genes HLA-DR2, with HLA-DR2 being strongly correlated with antibody titers against 38-kDa Mtb protein (17), thus further motivating the identification of antibody-diagnostics to guide TB clinical care (18, 19). However, like many infectious diseases, including COVID-19 (20, 21), antibody titers to Mtb increase with pathogen burden (22–25). Because antigen load can vary drastically across subjects, antibody magnitude may be an incomplete marker of disease progression. However, emerging data strongly suggest that antibodies differ across ATB and LTB, both with respect to antigen-specificities targeted selectively across disease states, but also with respect to differences in the quality of the Mtb-specific humoral immune response (isotype, subclass, and Fc-glycosylation) (26–29). Thus far, studies aiming at developing antibody-based diagnostics for ATB and LTB have focused on a small number of Mtb antigens including lipoarabinomannan (LAM), purified protein derivative (PPD), ESAT6/CFP10, Ag85A/B and MPT64 (26, 27, 30). Even in the setting of HIV, which is associated with CD4 T cell suppression and altered humoral immunity (4), humoral immune responses to Mtb have been shown to discriminate between ATB and LTB (30). However, whether additional Mtb antigen specificities and antibody qualities could provide clearer resolution of LTB and ATB remains unclear. Thus, in this study we comprehensively profiled the humoral immune response across 209 Mtb antigens, predicted to be highly enriched in the lung under hypoxic conditions as would be present in a granuloma (31, 32). Using a systems serology approach, we identified novel classes of antigen-specific antibody profiles able to resolve and discriminate ATB and LTB, independent of HIV status.

## Materials and Methods

### Study Subjects

Plasma samples from 4 groups of adults were included in this study: ATB/HIV+ (n = 12), LTB/HIV+ (n = 22), ATB/HIV- (n = 21), and LTB/HIV- (n = 22) (**Table 1**). All subjects were recruited from Cape Town, South Africa. LTB was defined as the absence of TB symptoms, no previous history of TB diagnosis or treatment and presence of IFN-γ based on an IFN-γ release assay (IGRA). ATB was defined as a positive culture for *Mycobacterium tuberculosis* (Mtb) growth or positive sputum smear microscopy. Blood from ATB individuals was obtained between 0 and 7 days of standard course anti-TB treatment following South African National Health Guidelines. None of the patients included in the study were on antiretroviral therapy (ART) at the time of enrollment. For each participant, whole blood was collected in sodium heparin Vacutainer tubes (BD Biosciences). Plasma samples were isolated by centrifugation under 500g after 5 minutes, within 4 hours of collection. Prior to the study, a written and informed consent was given by all study participants. Consents were approved by the Human Research Ethics Committee of the University of Cape Town and the Western Cape Department of Health, as well as the study institutional review board at Massachusetts General Hospital and Partners Healthcare.

**Table 1:** Demographic data and HIV-associated parameters.

	HIV+/ATB	HIV+/LTB	HIV-/ATB	HIV-/LTB
**Total number**	12	22	21	22
**Mean age (years ± SD)**	34,8 ± 7.6	32,8 ± 7.4	40,2 ± 10.5	28,6 ± 8.6
**Gender (Females)**	6 (50%)	17 (77%)	8 (38%)	9 (41%)
**Viral load mean (copies/ml ± SD)**	127722,5 ± 280219,9	38225,3 ± 62464,65		
**CD4+ T cell count mean** **(cells/mm^3^ ± SD)**	206,2 ± 183,6	506,8 ± 295,0		

### Antigens

Two hundred and nine Mtb antigens were used in this project. Purified LAM was received from BEI Resources and purified protein derivative (PPD) was obtained from the Statens Serum Institute. The remaining 207 proteins were recombinantly expressed Mtb antigens received from Dr. Tom Ottenhoff and Kees Franken (**Table S1**), and prepared as described previously (33, 34). These antigens were selected based on their immunogenicity and their discriminatory potential for TB diagnosis (33, 35, 36).

### Luminex Beads Coupling and Antigen-Specific Immunoglobulin Quantification

Antigen-specific antibody subclass and isotype levels present in the plasma of individuals with TB were measured using a custom multiplexed Luminex assay, as previously described (37). This customized Luminex platform has been used extensively across diseases (38–41). Mtb antigens were coupled to magnetic carboxylated fluorescent Luminex beads (Luminex Corporation) by carbodiimide-NHS ester coupling, with one bead region per antigen. Before the coupling with the antigens, beads were activated in an activation buffer containing 100 mM monobasic sodium phosphate (pH 6.2), in addition to 50 mg/ml N-hydroxysulfosuccinimide (Sulfo-NHS; Pierce) resuspended in H_2_0 and 1-ethyl-3-[3-dimethlyaminopropyl]carbodiimide-HCl (EDC; Pierce) resuspended in activation buffer. After a 30-minute incubation at room temperature (RT), beads were washed in coupling buffer (50 mM morpholineethanesulfonic acid (MES; pH 5.0)), then incubated with Mtb antigens for 2 hours at RT. Beads were then blocked during a 30 minute incubation at RT in phosphate buffered saline (PBS)-TBN (0.1% bovine serum albumin [BSA], 0.02% Tween 20, and 0.05% azide [pH 7.4]). Finally, beads coupled to proteins were washed in PBS-Tween (0.05% Tween 20) and stored in PBS with 0.05% sodium azide at 4°C. As LAM is a glycolipid, a modification by COOH-4-(4,6- dimethoxy[1,3,5]triazin-2-yl)-4-methyl-morpholinium (DMTMM) was required before the coupling to Luminex beads, as described previously (42). For this protocol, 2 μl of DMTMM (200 mg/ml; Sigma-Aldrich) were used for 25 μg of LAM. After 1 hour of incubation at RT, the excess of DMTMM was removed by using Sephadex G-25 PD-10 desalting columns (GE Healthcare). LAM was then added to the beads and incubated overnight at RT. The next day, LAM-coupled beads were washed in PBS then stored in PBS with 0.05% sodium azide at 4°C.

Antigen-coupled beads were added to plasma samples from TB individuals at 1:100 dilution in PBS and incubated at 4°C for 18 hours of shaking. Beads were then washed 3 times with PBS-Tween (0.05% Tween 20) and incubated with phycoerythrin (PE)-conjugated mouse anti-human IgG1, IgG3, IgA1 or IgM (Southern Biotech) at 1.3 μg/ml. After 1 hour of incubation at RT with shaking at 800 rpm, beads were washed 3 times with PBS-Tween (0.05% Tween 20) and resuspended in sheath fluid (Luminex Corporation). Each plasma sample was tested in duplicate, and PE median fluorescence intensity (MFI) levels were measured *via* the iQue screener plus (Intellicyt) analyzer.

### Antigen-Specific Fcγ-Receptor Binding

Fcγ-receptors (FcγRs) were purchased from Duke Human Vaccine Institute to study the relative binding levels of Mtb-specific antibodies to individual FcγRs (43). Avi-tagged FcγR2AR, FcγR2B, FcγR3AV and FcγR3B were biotinylated with a BirA biotin-protein ligase (BirA500; Avidity) and the excess of biotin was removed with Zeba spin desalting columns (7K MWCO; Thermo Fisher Scientific).

Diluted plasma samples (1:100) were added to antigen-coupled beads to form immune complexes as described above, and after 18 hours of incubation at 4°C, beads were washed 3 times with PBS-Tween (0.05% Tween 20). At the end of the incubation, streptavidin-R-phycoerythrin (ProZyme) was added to each biotinylated FcγRs in a 4:1 molar ratio during an incubation time of 20 minutes at RT. Fluorescent labeled FcγRs (1 μg/ml in 0.1% BSA-PBS) were then added to immune complexes and incubated for 1 hour at RT. Finally, beads were washed 3 times with PBS-Tween (0.05% Tween 20), then resuspended in sheath fluid (Luminex Corporation) and the median PE intensity was measured *via* the iQue screener plus (Intellicyt) system. Samples were tested in duplicate.

### Statistical and Computational Analysis

Data analysis was performed using R version 4.0.2 (2020-06-22). Comparisons between active and latent individuals were performed using a Mann-Whitney U-test test followed by a Benjamini-Hochberg (BH) correction for multiple comparisons.

A multivariate approach, combining a least absolute shrinkage and selection operator (LASSO) for feature selection and classification using partial least square discriminant analysis (PLS-DA) with the LASSO-selected features was used to define feature that discriminated active and latent antibody profiles. Prior to analysis, mean fluorescence (MFI) values were first log-transformed and all data were normalized using z-scoring. Models were built using the R package “ropls” version 1.20.0 (44) and “glmnet” version 4.0.2. Model accuracy was assessed using five-fold cross-validation. For each test fold, LASSO-based feature selection was repeated 100 times, features selected at least 90 times out of 100 were identified as selected features. A PLS-DA classifier was applied to the training set using the selected features, and a prediction accuracy was recorded. The model was also validated *via* permutation testing, where the performance of the selected model was evaluated against randomly shuffled active-latent labels. Selected features were ordered according to their Variable Importance in Projection (VIP) score, and the first two latent variables (LVs) of the PLS-DA model were used to visualize the samples.

## Results

### A Limited Mtb Antigen Panel Highlights Differences Across ATB and LTB but Does Not Fully Discriminate Across Disease Status

**Figure 1:**
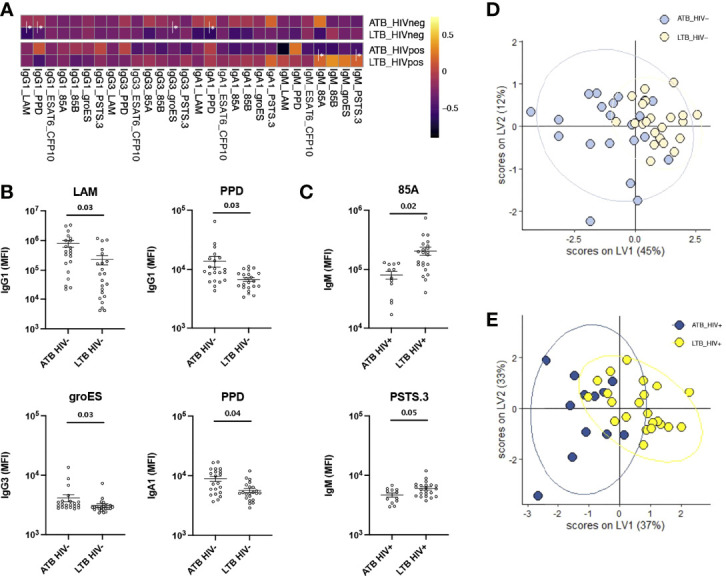
Antibody levels against common Mtb antigens differ between ATB and LTB patients. Relative levels of IgG1, IgG3, IgA1 and IgM against 8 common *Mtb* antigens (LAM, PPD, ESAT6/CFP10, Ag85A and 85B, groES and PSTS3) were quantified *via* Luminex in the plasma of ATB (n=21) and LTB (n=22) patients in the HIV negative group and ATB (n=12) and LTB (n=22) patients in the HIV positive group. **(A)** For the heatmap, Z-score transformation of the MFI values was performed, and the median of each group was graphed. Antibody levels that are significantly different between ATB and LTB are graphed separately for all individuals in the HIV negative **(B)** and the HIV positive **(C)** groups. A Mann-Whitney U-test test followed by a Benjamini-Hochberg (BH) correction for multiple comparisons was used to test for statistically significant differences ATB and LTB. Multivariate analysis using LASSO and PLS-DA model shows antibody levels comparisons between ATB and LTB in HIV negative **(D)** and positive **(E)** populations. Cross-validation accuracy for **(D, E)** was 62.8% and 67.6%, respectively.

To explore humoral responses in individuals with LTB and ATB, we began by profiling isotype and subclass levels across 8 common Mtb antigens (LAM, PPD, ESAT6/CFP10, Ag85A and 85B, groES and PSTS3) in ATB and LTB individuals that were HIV positive or negative. As previously described, HIV negative ATB individuals exhibited higher levels of IgG1, IgG3 and IgA1 responses to particular antigens, including LAM, PPD, and groES (**Figures 1A, B**). Conversely, an opposite profile was observed in HIV positive individuals, marked by significantly elevated humoral immune responses, and particularly IgM responses to Ag85A and PSTS3 among HIV positive LTB (**Figures 1A, C**). However, these features did not result in complete discrimination of ATB and LTB patients across HIV serostatus (**Figures 1D, E**) (cross-validation accuracy of 62.8% for HIV-; 67.6% for HIV+), suggesting that common antigen-specific antibody profiles provide only a modest level of discrimination across TB infection and disease states.

### An Expanded Mtb Antigen Panel Identifies Unappreciated Mtb-Specific Antibody Differences Between ATB and LTB Across HIV Infection Status

**Figure 2:**
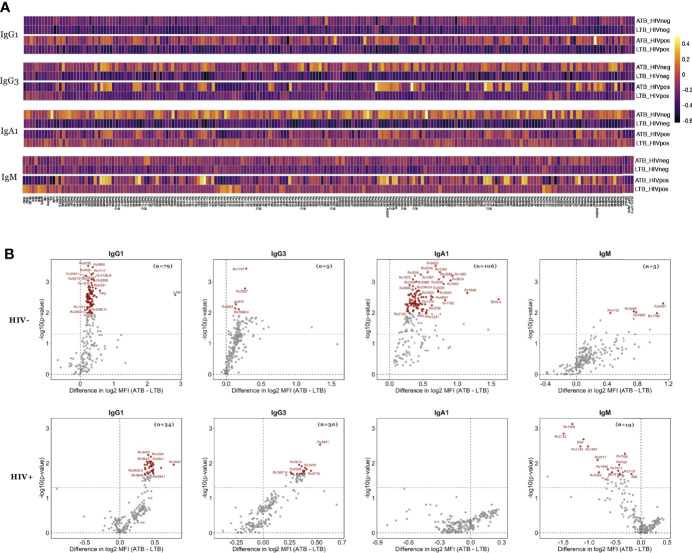
Mtb-specific antibody levels differ between ATB and LTB in HIV negative and HIV positive populations. Relative levels of IgG1, IgG3, IgA1 and IgM against 209 Mtb antigens were quantified *via* Luminex in the plasma of ATB (n=21) and LTB (n=22) patients in the HIV negative group and ATB (n=12) and LTB (n=22) patients in the HIV positive group. **(A)** Heatmaps illustrate the median of Z-scored MFI data for each group indicated. **(B)** The volcano plots characterize the magnitude (log2 fold change of ATB/LTB) and the significance (*p* values) of antibody levels between ATB and LTB. Values above black dashed lines are statistically different between ATB and LTB (*p* < 0.05). For adjusted *p* values, significant data are shown in red, non-significant differences are in black.

Despite the lack of differences across a limited number of common Mtb antigens (**Figure 1**), Mtb can express up to 4000 distinct antigens (45, 46), many of which may serve as additional antibody targets. Thus, we next elected to deeply profile the humoral immune response across LAM, PPD as well as 207 Mtb antigens (**Table S1**), transcriptionally enriched in *in vitro* or *in vivo* infection models, including lung specimens (36). Specifically, IgG1, IgG3, IgA1 and IgM levels were profiled across all antigens in ATB and LTB among HIV positive and HIV negative individuals (**Figure 2**). The data highlighted generally expanded IgG1 and IgG3 responses across HIV negative and positive ATB compared to LTB, with some sporadic elevated IgG1/IgG3 responses in HIV-positive LTB (**Figure 2A**). IgA responses were significantly elevated in HIV negative ATB compared to LTB, despite expanded IgA immunity in HIV-positive LTB (**Figure 2A**). IgM responses were more diffuse across HIV negative groups, despite the presence of a few high IgM responses in ATB compared to HIV negative LTB. Conversely, the IgM response was distinct in the setting of HIV co-infection, marked by significant expansions of particular Mtb-specific IgM responses that were either enriched in ATB or were enriched in LTB (**Figure 2A**). Thus, using this expanded antigen set, striking differences were noted in the evolution of the humoral immune response across HIV-serostatus, pointing to the presence of particular antigen-specific humoral immune responses that may discriminate between TB disease states.

### Minimal Sets of Mtb-Specific Antibody Responses Discriminate Between ATB and LTB Across HIV Status

To define the minimal set of specific antigens that were differentially targeted across the groups, we began by quantifying the number of antigens that were differentially targeted by each subclass or isotype of antibodies (**Table S2**). Specifically, in HIV negative individuals, 79 Mtb antigens were differentially targeted by IgG1, 5 antigens were differentially targeted by IgG3, 106 were differentially targeted by IgA1, and 5 antigens were targeted distinctly by IgM (**Figure 2B** and **Table S2**). Conversely, in HIV positive individuals, fewer statistically significant differences were observed after multiple correction but included: 34 Mtb antigens that were targeted statistically differently by IgG1, 30 that were differentially targeted by IgG3, and 19 antigens were targeted uniquely by IgM across the two groups (**Figure 2B**). Interestingly, for most comparisons, IgG1, IgG3, and IgA1 levels were higher in ATB individuals, in accordance with published data and likely reflective of the enhanced inflammatory state and bacterial burden observed in ATB infection among HIV positive individuals (22, 23). However, Mtb-specific IgM responses were targeted differently in HIV positive and negative individuals, with higher Mtb-specific IgM responses in ATB in the HIV negative group, but higher Mtb-specific IgM responses in LTB in the HIV positive population, reflecting a shift in B cell response profiles in the setting of HIV. Yet, collectively, these data highlight the striking differences in Mtb-specific humoral immune responses in the setting of HIV infection, but still associated to the persistence of differential humoral immune responses across ATB and LTB.

### Differential Mtb-Specific Antibody Binding to Fcγ-Receptors Across Mtb Disease States

**Figure 3:**
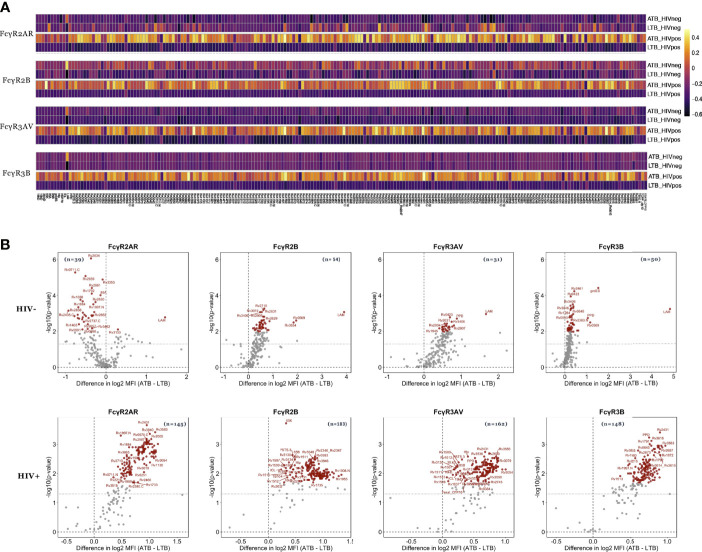
Mtb-specific FcR binding distinguishes ATB from LTB among HIV negative and positive individuals. The ability of antibodies to bind to FcgR2AR, FcgR2B, FcgR3AV and FcgR3B was measured in the plasma of ATB (n=21) and LTB (n=22) patients without HIV infection as well as ATB (n=12) and LTB (n=22) patients with HIV. **(A)** Heatmaps show median values of FcR binding after Z-score transformation to each of the 209 Mtb antigens. **(B)** Fold change between FcR binding in ATB and LTB as well as the significance (*p* values) of differences are plotted the volcano plots. Values above black dashed lines are statistically different between ATB and LTB (*p* < 0.05). For adjusted *p* values, significant data are shown in red, non-significant differences are in black.

Beyond overall changes in antibody subclass and isotype responses to Mtb, emerging data point to significant differences in the inflammatory state of Mtb-specific antibodies across disease states (26, 29). These changes are induced by alterations in antibody-Fc-glycosylation, aimed at deploying antibody effector functions required for enhanced clearance and control of the pathogen (47). Because these Fc-glycosylation changes result in altered binding to Fcγ-receptors (FcR), we next examined whether Mtb-specific FcR binding differences (FcγR2AR, FcγR2B, FcγR3AV, and FcγR3B) existed against LAM, PPD and 207 Mtb antigens (**Table S1**) in HIV positive and negative individuals with ATB or LTB (**Figure 3**).

Thirty-nine Mtb antigens were differentially recognized by FcγR2AR binding antibodies in HIV negative LTB populations compared to ATB (**Figure 3B**). Moreover, 54, 31 and 50 Mtb antigens were differentially recognized by FcγR2B, FcγR3AV and FcγR3B-binding antibodies, respectively, with higher binding observed in ATB (**Figures 3A, B** and **Table S2**). Conversely, FcR binding antibodies were globally shifted in HIV positive individuals, marked by disproportionately higher levels of FcγR2AR, FcγR2B, FcγR3AV and FcγR3B binding antibodies in HIV+ ATB compared to LTB (**Figure 3**). Specifically, 145, 183, 162 and 148-Mtb-antigens were targeted more robustly by FcγR2AR, FcγR2B, FcγR3AV and FcγR3B binding antibodies, respectively, in ATB compared to LTB (**Table S2**). Thus, beyond alterations in overall antibody subclass/isotype binding profiles across TB disease status, alterations are observed with FcR binding, that are amplified in the setting of HIV co-infection, again pointing to the possibility for antibody-based discrimination across LTB and ATB across HIV status.

### A Minimal Set of Antibody Biomarkers Can Discriminate LTB and ATB Irrespective of HIV Status

**Figure 4:**
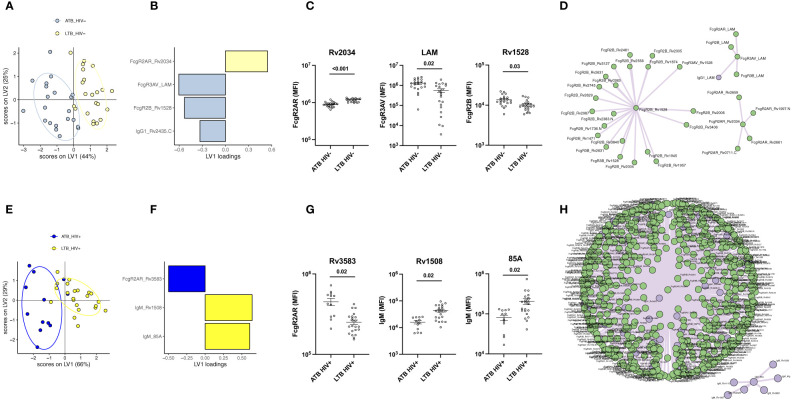
Individuals with ATB and LTB exhibit distinct humoral profile against Mtb antigens regardless of HIV infection. Multivariate analysis comparing antibody response in ATB and LTB among HIV negative **(A, B)** and HIV positive **(E, F)** populations. **(A, E)** PLS-DA models showing dot plots of nearly non-overlapping antibody features in ATB (blue) and LTB (yellow) from HIV negative **(A)** and positive **(E)** groups. Ellipses show 95% confidence intervals. PLDS-DA models were trained using LASSO-selected features that are plotted on VIP plots for HIV negative **(B)** and positive **(F)** patients. Cross-validation accuracy for **(A, E)** was 74.2% and 79.1%, respectively. Antibody features showing significant differences between ATB and LTB are graphed for the HIV negative **(C)** and HIV positive **(G)** cohorts. A Mann-Whitney U-test test followed by a Benjamini-Hochberg (BH) correction for multiple comparisons was used to test for statistically significant differences ATB and LTB. Network correlations depicting the additional non-LASSO-selected features that were correlated with the LASSO-selected parameters statistically different between ATB and LTB in the HIV negative **(D)** and positive **(H)** groups. Data on antibody levels are in purple, features related to FcγR binding are in green.

Given the significant differences in isotype, subclass, and FcR binding differences across LTB and ATB in both HIV positive and negative individuals, we next aimed to identify a minimal set of potential candidate antigens that could be used to discriminate between ATB and LTB. A computational analysis that combined LASSO feature down-selection and PLS-DA classification was conducted in the HIV negative (**Figures 4A–D**) and positive (**Figures 4E–H**) groups. Out of the 1680 antigen-specific antibody features included in the analysis, only 4 features were sufficient to separate HIV negative ATB and LTB (**Figures 4A, B**): RV2034-specific FcγR2AR binding, which was higher in LTB, as well as LAM-specific FcγR3A binding antibodies, RV1528-specific FcγR2B binding antibodies, and RV2435c IgG1 levels that were enriched in ATB (**Figure 4B**). Three of the LASSO selected features were significantly differentially targeted across the groups, including RV2034-specific FcγR2AR binding, LAM-FcγR3A binding and RV1528-specific FcγR2B binding antibodies (**Figure 4C**). Given that LASSO selects a minimal set of features that account for the greatest variance across the groups, the LASSO-selected features may represent groups of correlated antibody features that diverge across TB groups. Thus, to gain enhanced insights into the global changes across groups, a co-correlate analysis was built on the LASSO-selected features highlighting the presence of a large, expanded FcγR2B network in ATB, as well as two smaller networks of LAM-biomarkers and FcγR2AR binding biomarkers enriched in HIV negative ATB individuals (**Figure 4D**).

Similar to the HIV negative LTB/ATB individuals, as few as 3 of the 1680 analyzed features were sufficient to separate LTB and ATB individuals with HIV (**Figures 4E, F**). Specifically, RV3583 FcγR2AR binding was enriched in ATB individuals, and RV1508 and Ag85A IgM levels were enriched among LTB (**Figures 4E, F**). All features were significantly different across the groups (**Figure 4G**). Interestingly, co-correlates analysis revealed that the single ATB feature was tightly linked to nearly all IgG/FcR binding levels that were globally expanded in ATB (**Figure 4H**). Additionally, a smaller network of IgM features emerged, marking the unique expansion of IgM responses among HIV positive LTB.

**Figure 5:**
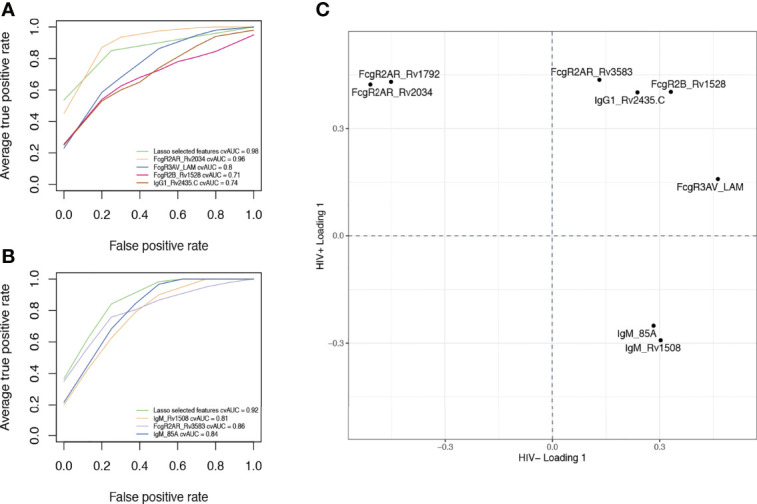
Discriminatory features between ATB and LTB. ROC curves showing the sensitivity and the specificity of potential biomarkers discriminating ATB and LTB in HIV negative **(A)** and positive **(B)** populations. **(C)** Loading plot showing LASSO-selected features enriched in ATB, both among HIV negative and HIV positive individuals (upper right quadrant) versus those enriched in LTB among HIV negative and HIV positive groups (low-left quadrant). Features in the upper left quadrant are increased in ATB compared to LTB among HIV+ but are enriched in LTB among HIV-. Features in the low right quadrant are enriched in LTB compared to ATB among HIV+ but are higher in ATB among HIV-.

Finally, we aimed to estimate the classification accuracy of the LASSO-selected antibody features across ATB and LTB. An area under the receiver operating characteristic (ROC) curve (AUC) was calculated for each feature (**Figures 5A, B**). Importantly, the combination of the selected antibody features gave an AUC close to 1 (AUC = 0.98 for HIV-; AUC = 0.92 for HIV+). However, even using individual antibody features, classification accuracies of 0.96, 0.8, 0.71 and 0.74 were observed with Rv2034-specific binding to FcγR2AR, LAM-specific binding to FcγR3AV, Rv1528-specific binding to FcγR2B and Rv2435C-specific IgG1 levels, respectively, among HIV negative individuals (**Figure 5A**). Similarly, in HIV positive individuals, individual feature AUCs reached 0.81, 0.86 and 0.84 for Rv1508-specific IgM levels, binding of Rv3583-specific antibodies to FcγR2AR and Ag85A-specific IgM levels, respectively (**Figure 5B**). Our results showed that 4 parameters were systematically enriched in ATB compared to LTB among HIV negative and positive populations, which are IgG1 levels against Rv2435.C, as well as Rv3583-, Rv1528- and LAM-binding to FcγRAR, FcγR2B and FcγR3AV, respectively (**Figure 5C**). Thus, a minimal set of largely novel Mtb-specific humoral biomarkers may provide a unique opportunity to help discriminate between LTB and ATB. Given the ease of generation of rapid point-of-care antibody-based diagnostics, these data point to a simple opportunity to develop rapid point-of-care tests for the diagnosis of Mtb disease state.

## Discussion

The lack of rapid and specific point-of care diagnostic tools has impeded the control of tuberculosis, particularly among HIV positive individuals (WHO report 2020; (4, 48, 49). Serum antibody-based tests represent promising alternatives to the current medically intensive diagnostic approaches associated to sputum collection for culture or non-specific tests like TST or IGRA to diagnose active TB. However, previous work with strict quantitative measurements of canonical antigen-specific antibody levels has failed, in the past, to identify Mtb-specific humoral biomarkers able to discriminate ATB and LTB (50). We thus aimed to broaden the scope of biomarker discovery, integrating both qualitative differences in humoral immune profiles as well as the breadth of antigen-specific antibody responses across individuals with/without TB among HIV positive and negative adults. In addition to the 8 common Mtb antigens (LAM, PPD, ESAT6/CFP10, Ag85A and 85B, groES and PSTS3), 201 additional novel Mtb antigens were included, collectively providing significant resolution of ATB and LTB in HIV negative populations, including higher levels of IgG1 against LAM and PPD in ATB individuals, as well as increased groES-specific IgG3 and PPD-specific IgA1 levels (**Figures 1**, **2**). Conversely, while HIV positive ATB/LTB were also resolvable, the antigen-specific antibody profiles used to discriminate these groups shifted to include higher 85A- and PSTS3-specific IgM response in LTB due to expected HIV induced alterations in immunity. This immune dysregulation in ATB patients with HIV might in part be attributable to lower CD4^+^ T cell counts and higher viral loads, resulting in altered B cell responses and elevated inflammation (**Table 1**). However, collectively, a set of antigen-specific antibody profiles were defined in both HIV negative and positive populations that provide antigen-specific resolution that could guide TB diagnostic development, providing a unique opportunity to build point-of-care diagnostic tools that would improve TB medical care.

Increased IgG and IgA responses have been previously observed in ATB individuals (22, 24, 30). Studies have shown that high titers are correlated with bacillary load in patient sputum (51). Interestingly, only a weak separation was observed between ATB and LTB when using a limited list of common Mtb antigens (accuracy: 62.8%) (**Figure 1**). Similarly, other studies have shown that antibodies targeting known antigens including Ag85, ESAT6, and CFP10 have limited diagnostic accuracy. Moreover, conflicting reports have demonstrated that the sensitivity of Ag85-specific IgG levels reaches 67.5% for the diagnosis of ATB (50), while others suggested that Ag85-specific IgG levels can provide up to 84.1% (52). Conversely, here the expansion of antigens to additional targets that may be expressed more abundantly in the lung during Mtb infection (36), improved diagnostic accuracy to 98% in HIV negative individuals and 92% in HIV positive subjects (**Figure 5**). Whether these antigen-specific antibodies contribute to the immune response to Mtb remains unclear but may reflect the presence of a more robust host (antibody) response to highly expressed antigens, that may more sensitively resolve individuals with differential control of Mtb.

Surprisingly, HIV co-infection abrogated the differences in IgG and IgA observed in ATB compared to LTB among HIV negative individuals. In accordance with our observations, data published previously reported that total amounts of LAM IgG were higher in ATB/HIV- compared to ATB/HIV+ individuals (30). Also, the loss of IgM observed in ATB individuals with HIV points to a significant shift in the humoral immune response upon co-infection (53). Moreover, given our emerging appreciation for the importance of IgM in Mtb control (54), it is plausible that the loss of IgM may compromise anti-microbial control in HIV/Mtb co-infection, contributing to disease progression. IgM is known for its antimicrobial function, inducing opsonophagocytosis, complement deposition and agglutination (55). Studies in mice have highlighted the main role that IgM plays in the control of Mtb infection by regulating immune response inside granulomas, leading to improved survival of infected mice during the chronic phase of the disease (56, 57). Here we observed that HIV positive individuals with LTB had higher IgM levels against particular Mtb antigens compared to individuals with ATB, including responses against Ag85A and PSTS3, potentially pointing to specific IgM targets that may represent critical Mtb therapeutic targets (**Figures 1**, **2**).

In addition to isotype differences across ATB/LTB, Mtb-specific FcR binding profiles were significantly differentiated across the groups, both in HIV positive and negative populations (**Figure 3**). Changes in FcR binding are attributable to both alterations in subclass distribution and Fc-glycosylation (58, 59), both of which shift rapidly in response to inflammatory cues that arise during infection. Thus, FcRs may act as sensitive markers of inflammatory changes in antibodies (47). Similarly, previous studies have highlighted striking differences in Fc-glycosylation across HIV negative LTB and ATB (26, 29). Moreover, despite the robust general Fc-inflammatory changes associated with HIV (60, 61), Fc-glycosylation changes still likely occur in the setting of HIV co-infection, marked by striking, and previously unappreciated highly significant increases in all FcR binding in the setting of ATB infection across most Mtb antigens (**Figure 3**). Interestingly, while the differences are not as pronounced in HIV negative ATB/LTB, Mtb-specific FcR measurements were still key to resolving the groups. FcR binding differences are likely attributed to significant changes in Fc-glycosylation, that can be detected using Mass-spectrometry, but may be more readily quantified, in a simpler point-of-care approach using FcR detection.

Specific antigen-dependent antibody responses were key to discriminating ATB and LTB. In HIV negative individuals, IgG1 levels against Rv2435.C as well as FcγR2AR, FcγR3AV and FcγR2B binding associated with Rv2034, LAM and Rv1528 respectively were sufficient for the separation of the groups (**Figure 4B**). In coinfected individuals, only 3 parameters were enough for ATB versus LTB discrimination: Rv3583-specific binding to FcγR2AR in addition to Rv1508- and Ag85A-specific IgM levels (**Figure 4F**). Other than LAM and Ag85A, the additional targets are less well defined. Rv2034 has been described as a transcriptional regulator form the ArsR family (62) and shown to be expressed during pulmonary infection, inducing a strong T cell response (63), and clearly - now - also a robust antibody response. Little is known about Rv1529 and Rv2435.C, the former thought to be a polyketide-associated acyltransferase involved in lipid metabolism (64), and Rv2435.C has been defined as an adenylyl cyclase essential for Mtb survival (65, 66). In HIV positive individuals, antibodies to Rv3583, a transcriptional regulator (67), were among the most discriminatory Mtb responses. Finally, IgM responses to Ag85A and Rv1508, the latter involved in cell surface structures and transport (68), were highly discriminatory. Interestingly, 4 features were similarly enriched in ATB compared to LTB in both HIV positive and negative groups, including RV2435c IgG1 levels as well as Rv3583-, Rv1528- and LAM-binding antibodies to FcγRAR, FcγR2B and FcγR3AV, respectively (**Figure 5C**). While it remains unclear how BCG-vaccination alters these antibody responses across populations, these data suggest that by including a set of 7 antigens and 3 Fc-readouts in a multiplexed diagnostic test, it may be possible to develop a single set of antigens and Fc-detectors to distinguish LTB and ATB across HIV positive and negative populations.

Collectively, this work identifies a number of novel antibody targets in ATB and LTB that may help guide diagnostic development. Specifically, these data point to novel combinations of Mtb antigen specificities and antibody Fc-qualities in discriminating between ATB/LTB. Indeed, in addition to LAM, RV2435c, Rv3583 and Rv1528 were also essential for the discrimination of ATB and LTB regardless of HIV status. While several combinations of inflammatory cytokine or transcriptomic biomarkers have been proposed as new exploratory approaches to help discriminate between ATB and LTB, the development of a simple Mtb-antigen-specific antibody point of care diagnostic would provide simplicity and ease of diagnosis. Moreover, while a single Mtb-antigen antibody Fc feature that could discriminate across ATB/LTB in both HIV negative and positive individuals would be desirable, a single solution may not be possible. However, with the advent of novel, simple lateral flow diagnostics that can multiplex antigen and detectors, the combination of the 7 antigens with unique detectors could be easily developed, providing a simple diagnostic to rapidly determine ATB/LTB status in any particular population. Whether additional antigen-specificities, not probed here, could add additional resolution is possible, and warrants additional analysis. Moreover, further discriminatory antibody profile discovery in IGRA-negative individuals living in TB-endemic areas, among BCG-vaccinated individuals living outside of endemic regions, and analysis of LTB and ATB from disparate geographical regions will provide a better insight into the robustness of these antibody signatures. Additionally, future functional and Fc-glycosylation profiling may reveal whether these unique humoral signatures also track with differential control of the infection, which might lead to the identification of features in LTB individuals that are associated with high risk of progression to ATB. While the ultimate development of a TB diagnostic will require extensive validation across multiple populations globally, overall, our data expand the current knowledge of Mtb antigen-specific humoral immunity and highlights the need to deeply understand how humoral changes may be leveraged both in vaccine and diagnostic development.

## Data Availability Statement

The original contributions presented in the study are included in the article/**Supplementary Material**. Further inquiries can be directed to the corresponding author.

## Ethics Statement

The studies involving human participants were reviewed and approved by Human Research Ethics Committee of the University of Cape Town and the Western Cape Department of Health, and the study institutional review board at Massachusetts General Hospital and Partners Healthcare. The patients/participants provided their written informed consent to participate in this study.

## Author Contributions

GA and NN designed and conceived the research study. NN and KF performed the experiments. DC, EI, WJ, and NN analyzed the data. MK and WH contributed with study cohort administration and implementation. NN and GA wrote the manuscript, with contributions from DC, LD, BF, KF, CD, and TO. All authors contributed to the article and approved the submitted version.

## Conflict of Interest

GA is a founder and equity holder in Systems Seromyx and Leyden Labs. GA’s interests were reviewed and managed by MGH and Partners HealthCare in accordance with their conflict of interest policies.

The remaining authors declare that the research was conducted in the absence of any commercial or financial relationships that could be construed as a potential conflict of interest.

## Publisher’s Note

All claims expressed in this article are solely those of the authors and do not necessarily represent those of their affiliated organizations, or those of the publisher, the editors and the reviewers. Any product that may be evaluated in this article, or claim that may be made by its manufacturer, is not guaranteed or endorsed by the publisher.

## References

[1] Lin PL Ford CB Coleman MT Myers AJ Gawande R Ioerger T. Sterilization of Granulomas is Common in Active and Latent Tuberculosis Despite Within-Host Variability in Bacterial Killing. Nat Med (2014) 20(1):75–9. doi: 10.1038/nm.3412 PMC3947310 24336248

[2] Bade P Simonetti F Sans S Laboudie P Kissane K Chappat N. Integrative Analysis of Human Macrophage Inflammatory Response Related to Mycobacterium Tuberculosis Virulence. Front Immunol (2021) 12:668060. doi: 10.3389/fimmu.2021.668060 34276658 PMC8284339

[3] Pai M Behr MA Dowdy D Dheda K Divangahi M Boehme CC. Tuberculosis. Nat Rev Dis Primers (2016) 2:16076. doi: 10.1038/nrdp.2016.76 27784885

[4] Esmail H Riou C Bruyn ED Lai RP Harley YXR Meintjes G. The Immune Response to Mycobacterium Tuberculosis in HIV-1-Coinfected Persons. Annu Rev Immunol (2018) 36:603–38. doi: 10.1146/annurev-immunol-042617-053420 29490165

[5] Furin J Cox H Pai M. Tuberculosis. Lancet (2019) 393(10181):1642–56. doi: 10.1016/S0140-6736(19)30308-3 30904262

[6] Pai M Denkinger CM Kik SV Rangaka MX Zwerling A Oxlade O. Gamma Interferon Release Assays for Detection of Mycobacterium Tuberculosis Infection. Clin Microbiol Rev (2014) 27(1):3–20. doi: 10.1128/CMR.00034-13 24396134 PMC3910908

[7] Lewinsohn DM Leonard MK LoBue PA Cohn DL Daley CL Desmond E. Official American Thoracic Society/Infectious Diseases Society of America/Centers for Disease Control and Prevention Clinical Practice Guidelines: Diagnosis of Tuberculosis in Adults and Children. Clin Infect Dis (2017) 64(2):111–5. doi: 10.1093/cid/ciw778 PMC5504475 28052967

[8] Tomaras GD Yates NL Liu P Qin L Fouda GG Chavez LL. Initial B-Cell Responses to Transmitted Human Immunodeficiency Virus Type 1: Virion-Binding Immunoglobulin M (IgM) and IgG Antibodies Followed by Plasma Anti-Gp41 Antibodies With Ineffective Control of Initial Viremia. J Virol (2008) 82(24):12449–63. doi: 10.1128/JVI.01708-08 PMC2593361 18842730

[9] Ivanyi J. Serodiagnosis of Tuberculosis: Due to Shift Track. Tuberculosis (Edinb) (2012) 92(1):31–7. doi: 10.1016/j.tube.2011.09.001 21930430

[10] Ciupe SM Ribeiro RM Perelson AS. Antibody Responses During Hepatitis B Viral Infection. PloS Comput Biol (2014) 10(7):e1003730. doi: 10.1371/journal.pcbi.1003730 25078553 PMC4117427

[11] Ivanyi J Krambovitis E Keen M. Evaluation of a Monoclonal Antibody (TB72) Based Serological Test for Tuberculosis. Clin Exp Immunol (1983) 54(2):337–45. PMC1535890 6197217

[12] Bothamley G Udani P Rudd R Festenstein F Ivanyi J. Humoral Response to Defined Epitopes of Tubercle Bacilli in Adult Pulmonary and Child Tuberculosis. Eur J Clin Microbiol Infect Dis (1988) 7(5):639–45. doi: 10.1007/BF01964242 2461861

[13] Chandramuki A Bothamley GH Brennan PJ Ivanyi J. Levels of Antibody to Defined Antigens of Mycobacterium Tuberculosis in Tuberculous Meningitis. J Clin Microbiol (1989) 27(5):821–5. doi: 10.1128/jcm.27.5.821-825.1989 PMC267436 2501348

[14] Wilkins EG Ivanyi J. Potential Value of Serology for Diagnosis of Extrapulmonary Tuberculosis. Lancet (1990) 336(8716):641–4. doi: 10.1016/0140-6736(90)92144-7 1975849

[15] Kozakiewicz L Phuah J Flynn J Chan J. The Role of B Cells and Humoral Immunity in Mycobacterium Tuberculosis Infection. Adv Exp Med Biol (2013) 783:225–50. doi: 10.1007/978-1-4614-6111-1_12 PMC4184189 23468112

[16] Achkar JM Chan J Casadevall A. B Cells and Antibodies in the Defense Against Mycobacterium Tuberculosis Infection. Immunol Rev (2015) 264(1):167–81. doi: 10.1111/imr.12276 PMC4629253 25703559

[17] Bothamley GH Beck JS Schreuder GM D’Amaro J de Vries RR Kardjito T. Association of Tuberculosis and M. Tuberculosis-Specific Antibody Levels With HLA. J Infect Dis (1989) 159(3):549–55. doi: 10.1093/infdis/159.3.549 2464654

[18] Ivanyi J Bothamley GH Jackett PS. Immunodiagnostic Assays for Tuberculosis and Leprosy. Br Med Bull (1988) 44(3):635–49. doi: 10.1093/oxfordjournals.bmb.a072273 3076811

[19] Wilkinson RJ Haslov K Rappuoli R Giovannoni F Narayanan PR Desai CR. Evaluation of the Recombinant 38-Kilodalton Antigen of Mycobacterium Tuberculosis as a Potential Immunodiagnostic Reagent. J Clin Microbiol (1997) 35(3):553–7. doi: 10.1128/jcm.35.3.553-557.1997 PMC229625 9041387

[20] Atyeo C Fischinger S Zohar T Slein MD Burke J Loos C. Distinct Early Serological Signatures Track With SARS-CoV-2 Survival. Immunity (2020) 53(3):524–532 e524. doi: 10.1016/j.immuni.2020.07.020 32783920 PMC7392190

[21] Bartsch YC Fischinger S Siddiqui SM Chen Z Yu J Gebre M. Discrete SARS-CoV-2 Antibody Titers Track With Functional Humoral Stability. Nat Commun (2021) 12(1):1018. doi: 10.1038/s41467-021-21336-8 33589636 PMC7884400

[22] Kunnath-Velayudhan S Davidow AL Wang HY Molina DM Huynh VT Salamon H. Proteome-Scale Antibody Responses and Outcome of Mycobacterium Tuberculosis Infection in Nonhuman Primates and in Tuberculosis Patients. J Infect Dis (2012) 206(5):697–705. doi: 10.1093/infdis/jis421 22732925 PMC3491745

[23] Coppola M Arroyo L van Meijgaarden KE Franken KL Geluk A Barrera LF. Differences in IgG Responses Against Infection Phase Related Mycobacterium Tuberculosis (Mtb) Specific Antigens in Individuals Exposed or Not to Mtb Correlate With Control of TB Infection and Progression. Tuberculosis (Edinb) (2017) 106:25–32. doi: 10.1016/j.tube.2017.06.001 28802401

[24] Lee JY Kim BJ Koo HK Kim J Kim JM Kook YH. Diagnostic Potential of IgG and IgA Responses to Mycobacterium Tuberculosis Antigens for Discrimination Among Active Tuberculosis, Latent Tuberculosis Infection, and Non-Infected Individuals. Microorganisms (2020) 8(7):1–16. doi: 10.3390/microorganisms8070979 PMC7409123 32629849

[25] Watson A Li H Ma B Weiss R Bendayan D Abramovitz L. Human Antibodies Targeting a Mycobacterium Transporter Protein Mediate Protection Against Tuberculosis. Nat Commun (2021) 12(1):602. doi: 10.1038/s41467-021-20930-0 33504803 PMC7840946

[26] Lu LL Chung AW Rosebrock TR Ghebremichael M Yu WH Grace PS. A Functional Role for Antibodies in Tuberculosis. Cell (2016) 167(2):433–443 e414. doi: 10.1016/j.cell.2016.08.072 27667685 PMC5526202

[27] Awoniyi DO Baumann R Chegou NN Kriel B Jacobs R Kidd M. Detection of a Combination of Serum IgG and IgA Antibodies Against Selected Mycobacterial Targets Provides Promising Diagnostic Signatures for Active TB. Oncotarget (2017) 8(23):37525–37. doi: 10.18632/oncotarget.16401 PMC5514927 28415587

[28] Lu LL Das J Grace PS Fortune SM Restrepo BI Alter G. Antibody Fc Glycosylation Discriminates Between Latent and Active Tuberculosis. J Infect Dis (2020) 222(12):2093–102. doi: 10.1093/infdis/jiz643 PMC7661770 32060529

[29] Grace PS Dolatshahi S Lu LL Cain A Palmieri F Petrone L. Antibody Subclass and Glycosylation Shift Following Effective TB Treatment. Front Immunol (2021) 12:679973. doi: 10.3389/fimmu.2021.679973 34290702 PMC8287567

[30] van Woudenbergh E Irvine EB Davies L de Kock M Hanekom WA Day CL. HIV Is Associated With Modified Humoral Immune Responses in the Setting of HIV/TB Coinfection. mSphere (2020) 5(3):1–19. doi: 10.1128/mSphere.00104-20 PMC7380575 32434838

[31] Prosser G Brandenburg J Reiling N Barry CE 3rd Wilkinson RJ Wilkinson KA. The Bacillary and Macrophage Response to Hypoxia in Tuberculosis and the Consequences for T Cell Antigen Recognition. Microbes Infect (2017) 19(3):177–92. doi: 10.1016/j.micinf.2016.10.001 PMC5335906 27780773

[32] Zenk SF Hauck S Mayer D Grieshober M Stenger S. Stabilization of Hypoxia-Inducible Factor Promotes Antimicrobial Activity of Human Macrophages Against Mycobacterium Tuberculosis. Front Immunol (2021) 12:678354. doi: 10.3389/fimmu.2021.678354 34149713 PMC8206807

[33] Franken KL Hiemstra HS van Meijgaarden KE Subronto Y den Hartigh J Ottenhoff TH. Purification of His-Tagged Proteins by Immobilized Chelate Affinity Chromatography: The Benefits From the Use of Organic Solvent. Protein Expr Purif (2000) 18(1):95–9. doi: 10.1006/prep.1999.1162 10648174

[34] Commandeur S van Meijgaarden KE Prins C Pichugin AV Dijkman K van den Eeden SJ. An Unbiased Genome-Wide Mycobacterium Tuberculosis Gene Expression Approach to Discover Antigens Targeted by Human T Cells Expressed During Pulmonary Infection. J Immunol (2013) 190(4):1659–71. doi: 10.4049/jimmunol.1201593 23319735

[35] Serra-Vidal MM Latorre I Franken KL Diaz J de Souza-Galvao ML Casas I. Immunogenicity of 60 Novel Latency-Related Antigens of Mycobacterium Tuberculosis. Front Microbiol (2014) 5:517. doi: 10.3389/fmicb.2014.00517 25339944 PMC4189613

[36] Coppola M Ottenhoff TH. Genome Wide Approaches Discover Novel Mycobacterium Tuberculosis Antigens as Correlates of Infection, Disease, Immunity and Targets for Vaccination. Semin Immunol (2018) 39:88–101. doi: 10.1016/j.smim.2018.07.001 30327124

[37] Brown EP Licht AF Dugast AS Choi I Bailey-Kellogg C Alter G. High-Throughput, Multiplexed IgG Subclassing of Antigen-Specific Antibodies From Clinical Samples. J Immunol Methods (2012) 386(1-2):117–23. doi: 10.1016/j.jim.2012.09.007 PMC3475184 23023091

[38] Brown EP Weiner JA Lin S Natarajan H Normandin E Barouch DH. Optimization and Qualification of an Fc Array Assay for Assessments of Antibodies Against HIV-1/SIV. J Immunol Methods (2018) 455:24–33. doi: 10.1016/j.jim.2018.01.013 29395167 PMC5851662

[39] Offersen R Yu WH Scully EP Julg B Euler Z Sadanand S. HIV Antibody Fc N-Linked Glycosylation Is Associated With Viral Rebound. Cell Rep (2020) 33(11):108502. doi: 10.1016/j.celrep.2020.108502 33326789

[40] Fischinger S Cizmeci D Shin S Davies L Grace PS Sivro A. A Mycobacterium Tuberculosis Specific IgG3 Signature of Recurrent Tuberculosis. Front Immunol (2021) 12:729186. doi: 10.3389/fimmu.2021.729186 34630406 PMC8493041

[41] Herman JD Wang C Loos C Yoon H Rivera J Dieterle ME. Functional Convalescent Plasma Antibodies and Pre-Infusion Titers Shape the Early Severe COVID-19 Immune Response. Nat Commun 12(1):6853. doi: 10.1038/s41467-021-27201-y PMC8617042 34824251

[42] Schlottmann SA Jain N Chirmule N Esser MT. A Novel Chemistry for Conjugating Pneumococcal Polysaccharides to Luminex Microspheres. J Immunol Methods (2006) 309(1-2):75–85. doi: 10.1016/j.jim.2005.11.019 16448665

[43] Brown EP Dowell KG Boesch AW Normandin E Mahan AE Chu T. Multiplexed Fc Array for Evaluation of Antigen-Specific Antibody Effector Profiles. J Immunol Methods (2017) 443:33–44. doi: 10.1016/j.jim.2017.01.010 28163018 PMC5333794

[44] Thevenot EA Roux A Xu Y Ezan E Junot C. Analysis of the Human Adult Urinary Metabolome Variations with Age, Body Mass Index, and Gender by Implementing a Comprehensive Workflow for Univariate and OPLS Statistical Analyses. J Proteome Res (2015) 14(8):3322–]35. doi: 10.1021/acs.jproteome.5b00354 26088811

[45] Geluk A van Meijgaarden KE Joosten SA Commandeur S Ottenhoff TH. Innovative Strategies to Identify M. Tuberculosis Antigens and Epitopes Using Genome-Wide Analyses. Front Immunol (2014) 5:256. doi: 10.3389/fimmu.2014.00256 25009541 PMC4069478

[46] Coppola M van Meijgaarden KE Franken KL Commandeur S Dolganov G Kramnik I. New Genome-Wide Algorithm Identifies Novel *In-Vivo* Expressed Mycobacterium Tuberculosis Antigens Inducing Human T-Cell Responses With Classical and Unconventional Cytokine Profiles. Sci Rep (2016) 6:37793. doi: 10.1038/srep37793 27892960 PMC5125271

[47] Irvine EB Alter G. Understanding the Role of Antibody Glycosylation Through the Lens of Severe Viral and Bacterial Diseases. Glycobiology (2020) 30(4):241–53. doi: 10.1093/glycob/cwaa018 PMC7109349 32103252

[48] Gupta RK Lucas SB Fielding KL Lawn SD. Prevalence of Tuberculosis in Post-Mortem Studies of HIV-Infected Adults and Children in Resource-Limited Settings: A Systematic Review and Meta-Analysis. AIDS (2015) 29(15):1987–2002. doi: 10.1097/QAD.0000000000000802 26266773 PMC4568896

[49] The Lancet Infectious D. Tuberculosis and Malaria in the Age of COVID-19. Lancet Infect Dis (2021) 21(1):1. doi: 10.1016/S1473-3099(20)30946-4 33357386 PMC7758173

[50] Raja A Uma Devi KR Ramalingam B Brennan PJ. Improved Diagnosis of Pulmonary Tuberculosis by Detection of Free and Immune Complex-Bound Anti-30 kDa Antibodies. Diagn Microbiol Infect Dis (2004) 50(4):253–9. doi: 10.1016/j.diagmicrobio.2004.08.010 15582298

[51] Jackett PS Bothamley GH Batra HV Mistry A Young DB Ivanyi J. Specificity of Antibodies to Immunodominant Mycobacterial Antigens in Pulmonary Tuberculosis. J Clin Microbiol (1988) 26(11):2313–8. doi: 10.1128/jcm.26.11.2313-2318.1988 PMC266883 2466869

[52] Kumar G Dagur PK Singh PK Shankar H Yadav VS Katoch VM. Serodiagnostic Efficacy of Mycobacterium Tuberculosis 30/32-kDa Mycolyl Transferase Complex, ESAT-6, and CFP-10 in Patients With Active Tuberculosis. Arch Immunol Ther Exp (Warsz) (2010) 58(1):57–65. doi: 10.1007/s00005-009-0055-4 20049651 PMC2816261

[53] De Milito A Nilsson A Titanji K Thorstensson R Reizenstein E Narita M. Mechanisms of Hypergammaglobulinemia and Impaired Antigen-Specific Humoral Immunity in HIV-1 Infection. Blood (2004) 103(6):2180–6. doi: 10.1182/blood-2003-07-2375 14604962

[54] Irvine EB O’Neil A Darrah PA Shin S Choudhary A Li W. Robust IgM Responses Following Intravenous Vaccination With Bacille Calmette-Guerin Associate With Prevention of Mycobacterium Tuberculosis Infection in Macaques. Nat Immunol (2021) 22(12):1515–23. doi: 10.1038/s41590-021-01066-1 PMC8642241 34811542

[55] Ehrenstein MR Notley CA. The Importance of Natural IgM: Scavenger, Protector and Regulator. Nat Rev Immunol (2010) 10(11):778–86. doi: 10.1038/nri2849 20948548

[56] Chan J Mehta S Bharrhan S Chen Y Achkar JM Casadevall A. The Role of B Cells and Humoral Immunity in Mycobacterium Tuberculosis Infection. Semin Immunol (2014) 26(6):588–600. doi: 10.1016/j.smim.2014.10.005 25458990 PMC4314354

[57] Ordonez C Savage HP Tarajia M Rivera R Weeks-Galindo C Sambrano D. Both B-1a and B-1b Cells Exposed to Mycobacterium Tuberculosis Lipids Differentiate Into IgM Antibody-Secreting Cells. Immunology (2018). doi: 10.1111/imm.12909 PMC6050208 29455451

[58] Bruhns P Iannascoli B England P Mancardi DA Fernandez N Jorieux S. Specificity and Affinity of Human Fcgamma Receptors and Their Polymorphic Variants for Human IgG Subclasses. Blood (2009) 113(16):3716–25. doi: 10.1182/blood-2008-09-179754 19018092

[59] Jennewein MF Alter G. The Immunoregulatory Roles of Antibody Glycosylation. Trends Immunol (2017) 38(5):358–72. doi: 10.1016/j.it.2017.02.004 28385520

[60] Moore JS Wu X Kulhavy R Tomana M Novak J Moldoveanu Z. Increased Levels of Galactose-Deficient IgG in Sera of HIV-1-Infected Individuals. AIDS (2005) 19(4):381–9. doi: 10.1097/01.aids.0000161767.21405.68 15750391

[61] Vadrevu SK Trbojevic-Akmacic I Kossenkov AV Colomb F Giron LB Anzurez A. Frontline Science: Plasma and Immunoglobulin G Galactosylation Associate With HIV Persistence During Antiretroviral Therapy. J Leukoc Biol (2018) 104(3):461–71. doi: 10.1002/JLB.3HI1217-500R PMC6113120 29633346

[62] Gao CH Yang M He ZG. Characterization of a Novel ArsR-Like Regulator Encoded by Rv2034 in Mycobacterium Tuberculosis. PloS One (2012) 7(4):e36255. doi: 10.1371/journal.pone.0036255 22558408 PMC3338718

[63] Commandeur S van den Eeden SJ Dijkman K Clark SO van Meijgaarden KE Wilson L. The *In Vivo* Expressed Mycobacterium Tuberculosis (IVE-TB) Antigen Rv2034 Induces CD4(+) T-Cells That Protect Against Pulmonary Infection in HLA-DR Transgenic Mice and Guinea Pigs. Vaccine (2014) 32(29):3580–8. doi: 10.1016/j.vaccine.2014.05.005 24837764

[64] Angala SK Belardinelli JM Huc-Claustre E Wheat WH Jackson M. The Cell Envelope Glycoconjugates of Mycobacterium Tuberculosis. Crit Rev Biochem Mol Biol (2014) 49(5):361–99. doi: 10.3109/10409238.2014.925420 PMC4436706 24915502

[65] Gazdik MA McDonough KA. Identification of Cyclic AMP-Regulated Genes in Mycobacterium Tuberculosis Complex Bacteria Under Low-Oxygen Conditions. J Bacteriol (2005) 187(8):2681–92. doi: 10.1128/JB.187.8.2681-2692.2005 PMC1070381 15805514

[66] Yesilkaya H Dale JW Strachan NJ Forbes KJ. Natural Transposon Mutagenesis of Clinical Isolates of Mycobacterium Tuberculosis: How Many Genes Does a Pathogen Need? J Bacteriol (2005) 187(19):6726–32. doi: 10.1128/JB.187.19.6726-6732.2005 PMC1251597 16166535

[67] Sarmadian H Nazari R Zolfaghari MR Pirayandeh M Sadrnia M Arjomandzadegan M. Study of carD Gene Sequence in Clinical Isolates of Mycobacterium Tuberculosis. Acta Microbiol Immunol Hung (2014) 61(1):1–10. doi: 10.1556/AMicr.61.2014.1.1 24631749

[68] Garnier T Eiglmeier K Camus JC Medina N Mansoor H Pryor M. The Complete Genome Sequence of Mycobacterium Bovis. Proc Natl Acad Sci USA (2003) 100(13):7877–82. doi: 10.1073/pnas.1130426100 PMC164681 12788972

