# Corrigendum: Defining discriminatory antibody fingerprints in active and latent tuberculosis

**DOI:** 10.3389/fimmu.2023.1329784

**Published:** 2023-11-21

**Authors:** Nadege Nziza, Deniz Cizmeci, Leela Davies, Edward B. Irvine, Wonyeong Jung, Brooke A. Fenderson, Marwou de Kock, Willem A. Hanekom, Kees L. M. C. Franken, Cheryl L. Day, Tom H. M. Ottenhoff, Galit Alter

**Affiliations:** ^1^ Ragon Institute of Massachusetts General Hospital (MGH), Massachusetts Institute of Technology (MIT) and Harvard, Cambridge, MA, United States; ^2^ Division of Infectious Diseases, Brigham and Women’s Hospital, Boston, MA, United States; ^3^ Department of Immunology and Infectious Diseases, Harvard T.H. Chan School of Public Health, Boston, MA, United States; ^4^ South African Tuberculosis Vaccine Initiative (SATVI) and School of Child and Adolescent Health, Institute of Infectious Diseases and Molecular Medicine, University of Cape Town, Cape Town, South Africa; ^5^ Africa Health Research Institute, Durban, South Africa; ^6^ Division of Infection and Immunity, University College London, London, United Kingdom; ^7^ Department of Infectious Disease, Leiden University, Leiden, Netherlands; ^8^ Department of Microbiology and Immunology, Emory University School of Medicine, Emory University, Atlanta, GA, United States

**Keywords:** active and latent tuberculosis, antibodies, HIV, biomarkers, diagnostics

In the published article, there was an error in Supplemental Table 1 as published. An incorrect antigen was added to the list: Rv1926. The corrected Supplemental Table 1, without Rv1926 and its caption “List of Mtb proteins” are attached.

The authors apologize for this error and state that this does not change the scientific conclusions of the article in any way. The original article has been updated.

