# Supplementary material for: Defining Discriminatory Antibody Fingerprints in Active and Latent Tuberculosis
Source: Front Immunol. 2022 Apr 20;13:856906. doi: 10.3389/fimmu.2022.856906 (PMC9066635; doi:10.3389/fimmu.2022.856906)
Supplement: Supplementary file 2 [file Table_2.docx]

Supplementary Material

**Table S2.** Mtb antigens associated with different antibody response between ATB and LTB.

| **IgG1** | | | | | |
| --- | --- | --- | --- | --- | --- |
| **HIV-** | | | **HIV+** | | |
| **Mtb antigens** | **log Fold Change**  **ATB/LTB** | **P-value** | **Mtb antigens** | **log Fold Change**  **ATB/LTB** | **P-value** |
| Rv2030 | 0.0710 | 0.0163 | Rv2324 | 0.1330 | 0.0275 |
| Rv0990 | 0.1112 | 0.0172 | Rv3420 | 0.1219 | 0.0275 |
| Rv0081 | 0.1013 | 0.0185 | Rv0501 | 0.1305 | 0.0290 |
| Rv1717 | 0.1149 | 0.0185 | Rv0642 | 0.1271 | 0.0290 |
| Rv2661 | 0.0527 | 0.0185 | Rv2982 | 0.1158 | 0.0290 |
| Rv1736-N | 0.1164 | 0.0194 | Rv3427 | 0.1347 | 0.0290 |
| Rv2631 | 0.1009 | 0.0194 | Rv0640 | 0.1200 | 0.0292 |
| Rv0573 | 0.0724 | 0.0198 | Rv1479 | 0.1417 | 0.0292 |
| Rv2659 | 0.1253 | 0.0198 | Rv2380m | 0.1314 | 0.0292 |
| Rv1812 | 0.0992 | 0.0225 | Rv0066 | 0.1451 | 0.0303 |
| Rv0331 | 0.1052 | 0.0239 | Rv0468 | 0.1359 | 0.0303 |
| Rv1945 | 0.0794 | 0.0240 | Rv1792 | 0.1067 | 0.0303 |
| Rv0574 | 0.1115 | 0.0248 | Rv2737-C | 0.1168 | 0.0303 |
| Rv2987 | 0.0735 | 0.0248 | Rv3583 | 0.2318 | 0.0303 |
| Rv3406 | 0.0878 | 0.0248 | Rv0470 | 0.1304 | 0.0311 |
| Rv0423 | 0.1388 | 0.0249 | Rv1284 | 0.1383 | 0.0311 |
| PPD | 0.1765 | 0.0254 | Rv2380-N | 0.1359 | 0.0311 |
| Rv0767 | 0.0859 | 0.0254 | Rv3048 | 0.1329 | 0.0311 |
| Rv2383-C | 0.0789 | 0.0254 | Rv1038 | 0.1253 | 0.0331 |
| Rv2982 | 0.0830 | 0.0254 | Rv1997-N | 0.1774 | 0.0331 |
| Rv3463 | 0.1152 | 0.0254 | Rv2431 | 0.1081 | 0.0331 |
| Rv3710 | 0.0923 | 0.0254 | Rv0383 | 0.1429 | 0.0356 |
| Rv0140 | 0.0861 | 0.0263 | Rv1390 | 0.1097 | 0.0356 |
| Rv0501 | 0.1046 | 0.0263 | Rv0824 | 0.1423 | 0.0377 |
| Rv1073 | 0.0981 | 0.0263 | Rv2461 | 0.1371 | 0.0377 |
| Rv1471 | 0.1051 | 0.0263 | Rv3052 | 0.1360 | 0.0377 |
| Rv1734 | 0.0928 | 0.0263 | Rv0645 | 0.1284 | 0.0408 |
| Rv2225 | 0.1060 | 0.0263 | Rv1221 | 0.1177 | 0.0408 |
| Rv2558 | 0.1024 | 0.0263 | Rv3865 | 0.1422 | 0.0408 |
| **HIV-** | | | **HIV+** | | |
| **Mtb antigens** | **log Fold Change**  **ATB/LTB** | **P-value** | **Mtb antigens** | **log Fold Change**  **ATB/LTB** | **P-value** |
| Rv3126 | 0.0657 | 0.0263 | Rv2435-C | 0.1078 | 0.0441 |
| Rv3133 | 0.0633 | 0.0263 | Rv2941 | 0.1441 | 0.0441 |
| LAM | 0.8530 | 0.0270 | Rv3846 | 0.1213 | 0.0441 |
| Rv2629 | 0.0732 | 0.0270 | Rv2225 | 0.1070 | 0.0471 |
| Rv0080 | 0.0580 | 0.0270 | Rv2346_Rv2347 | 0.1309 | 0.0471 |
| Rv2035 | 0.0775 | 0.0270 |  |  |  |
| Rv2324 | 0.1178 | 0.0270 |  |  |  |
| Rv2383-N | 0.0805 | 0.0270 |  |  |  |
| Rv2627 | 0.0585 | 0.0270 |  |  |  |
| Rv3048 | 0.1461 | 0.0270 |  |  |  |
| Rv3129 | 0.0639 | 0.0270 |  |  |  |
| Rv0571 | 0.0695 | 0.0280 |  |  |  |
| Rv0645 | 0.0885 | 0.0280 |  |  |  |
| Rv1284 | 0.0905 | 0.0280 |  |  |  |
| Rv1738 | 0.0605 | 0.0281 |  |  |  |
| Rv1955 | 0.1285 | 0.0281 |  |  |  |
| Rv0642 | 0.1053 | 0.0286 |  |  |  |
| Rv0839 | 0.0645 | 0.0286 |  |  |  |
| Rv2657 | 0.0826 | 0.0286 |  |  |  |
| Rv1875 | 0.0693 | 0.0301 |  |  |  |
| Rv2004 | 0.0786 | 0.0301 |  |  |  |
| Rv2737-C | 0.0555 | 0.0301 |  |  |  |
| Rv3846 | 0.1100 | 0.0301 |  |  |  |
| Rv1956 | 0.0981 | 0.0308 |  |  |  |
| Rv1957 | 0.0765 | 0.0308 |  |  |  |
| Rv1996 | 0.0733 | 0.0308 |  |  |  |
| Rv2028 | 0.0872 | 0.0308 |  |  |  |
| Rv3019 | 0.0640 | 0.0308 |  |  |  |
| Rv3128 | 0.0724 | 0.0308 |  |  |  |
| Rv3427 | 0.0903 | 0.0320 |  |  |  |
| Rv2461 | 0.1057 | 0.0323 |  |  |  |
| Rv2642 | 0.0924 | 0.0323 |  |  |  |
| Rv1479 | 0.1021 | 0.0331 |  |  |  |
| Rv1806 | 0.0966 | 0.0331 |  |  |  |
|  |  |  |  |  |  |
| **HIV-** | | | **HIV+** | | |
| **Mtb antigens** | **log Fold Change**  **ATB/LTB** | **P-value** | **Mtb antigens** | **log Fold Change**  **ATB/LTB** | **P-value** |
| Rv2941 | 0.1075 | 0.0331 |  |  |  |
| Rv3052 | 0.0941 | 0.0331 |  |  |  |
| Rv0383 | 0.0975 | 0.0334 |  |  |  |
| Rv3420 | 0.1193 | 0.0334 |  |  |  |
| Rv0570-C | 0.0682 | 0.0348 |  |  |  |
| Rv2624 | 0.0616 | 0.0348 |  |  |  |
| Rv3229 | 0.0811 | 0.0348 |  |  |  |
| Rv1872 | 0.0641 | 0.0374 |  |  |  |
| Rv1131 | 0.0438 | 0.0393 |  |  |  |
| Rv2380m | 0.0868 | 0.0393 |  |  |  |
| Rv1908-C | 0.0869 | 0.0409 |  |  |  |
| Rv0824 | 0.0998 | 0.0431 |  |  |  |
| Rv3307 | 0.0782 | 0.0431 |  |  |  |
| Rv2380-N | 0.1078 | 0.0448 |  |  |  |
| Rv3840 | 0.0659 | 0.0448 |  |  |  |
| Rv2913 | 0.0919 | 0.0480 |  |  |  |

| **IgG3** | | | | | |
| --- | --- | --- | --- | --- | --- |
| **HIV-** | | | **HIV+** | | |
| **Mtb antigens** | **log Fold Change**  **ATB/LTB** | **P-value** | **Mtb antigens** | **log Fold Change**  **ATB/LTB** | **P-value** |
| Rv1791 | 0.0769 | 0.0174 | Rv2431 | 0.1599 | 0.0265 |
| Rv2007 | 0.0808 | 0.0249 | Rv3615 | 0.1016 | 0.0303 |
| groES | 0.0418 | 0.0323 | Rv2324 | 0.1087 | 0.0311 |
| Rv2661 | 0.0385 | 0.0363 | Rv3420 | 0.1185 | 0.0331 |
| Rv1908-C | 0.0533 | 0.0374 | Rv2982 | 0.1225 | 0.0356 |
|  |  |  | Rv0423 | 0.1160 | 0.0377 |
|  |  |  | Rv0501 | 0.1163 | 0.0377 |
|  |  |  | Rv2710 | 0.1347 | 0.0377 |
|  |  |  | Rv2941 | 0.1124 | 0.0377 |
|  |  |  | Rv1479 | 0.1138 | 0.0408 |
|  |  |  | Rv1997-N | 0.0779 | 0.0408 |
|  |  |  | Rv0468 | 0.0957 | 0.0441 |
|  |  |  | Rv0824 | 0.1031 | 0.0441 |
|  |  |  | Rv1038 | 0.1090 | 0.0441 |
|  |  |  | Rv1980 | 0.0804 | 0.0441 |
|  |  |  | Rv2737-C | 0.1086 | 0.0441 |
|  |  |  | Rv3846 | 0.1163 | 0.0441 |
|  |  |  | Rv3865 | 0.0839 | 0.0441 |
|  |  |  | Rv0470 | 0.1019 | 0.0471 |
|  |  |  | Rv0640 | 0.0869 | 0.0471 |
|  |  |  | Rv1284 | 0.0952 | 0.0471 |
|  |  |  | Rv1791 | 0.1115 | 0.0471 |
|  |  |  | Rv1872 | 0.1147 | 0.0471 |
|  |  |  | Rv2245 | 0.1022 | 0.0471 |
|  |  |  | Rv2380-N | 0.0985 | 0.0471 |
|  |  |  | Rv2380m | 0.0967 | 0.0471 |
|  |  |  | Rv3048 | 0.1136 | 0.0471 |
|  |  |  | Rv3052 | 0.1117 | 0.0471 |
|  |  |  | Rv3583 | 0.0973 | 0.0471 |
|  |  |  | Rv3616 | 0.0657 | 0.0471 |

| **IgA1** | | | | | | | | |
| --- | --- | --- | --- | --- | --- | --- | --- | --- |
| **HIV-** | | | | | | **HIV+** | | |
| **Mtb**  **antigens** | | **log Fold Change**  **ATB/LTB** | | **P-value** | | **Mtb antigens** | **log Fold Change**  **ATB/LTB** | **P-value** |
| Rv0423 | | 0.2062 | | 0.0163 | |  |  |  |
| Rv1390 | | 0.2247 | | 0.0185 | |  |  |  |
| Rv1957 | | 0.1406 | | 0.0185 | |  |  |  |
| Rv1980 | | 0.2803 | | 0.0185 | |  |  |  |
| Rv2005 | | 0.1131 | | 0.0185 | |  |  |  |
| Rv2034 | | 0.1708 | | 0.0185 | |  |  |  |
| Rv1875 | | 0.1026 | | 0.0194 | |  |  |  |
| Rv2245 | | 0.2203 | | 0.0194 | |  |  |  |
| Rv2389 | | 0.2406 | | 0.0194 | |  |  |  |
| Rv3616 | | 0.2717 | | 0.0198 | |  |  |  |
| Rv2988 | | 0.1199 | | 0.0215 | |  |  |  |
| Rv3865 | | 0.2418 | | 0.0236 | |  |  |  |
| Rv0080 | | 0.0883 | | 0.0239 | |  |  |  |
| Rv2006 | | 0.1027 | | 0.0240 | |  |  |  |
| Rv0839 | | 0.1014 | | 0.0254 | |  |  |  |
| Rv0990 | | 0.1278 | | 0.0254 | |  |  |  |
| Rv2029 | | 0.2163 | | 0.0254 | |  |  |  |
| Rv3420 | | 0.1332 | | 0.0254 | |  |  |  |
| Rv0470 | | 0.1100 | | 0.0263 | |  |  |  |
| Rv1403 | | 0.1107 | | 0.0263 | |  |  |  |
| Rv1846 | | 0.3460 | | 0.0263 | |  |  |  |
| Rv2383-N | | 0.1163 | | 0.0263 | |  |  |  |
| Rv2643 | | 0.2171 | | 0.0263 | |  |  |  |
| Rv2659 | | 0.0946 | | 0.0263 | |  |  |  |
| Rv2737-C | | 0.0944 | | 0.0263 | |  |  |  |
| Rv2989 | | 0.1401 | | 0.0263 | |  |  |  |
| Rv3406 | | 0.1193 | | 0.0263 | |  |  |  |
| Rv3710 | | 0.0931 | | 0.0263 | |  |  |  |
| Rv0645 | | 0.1167 | | 0.0270 | |  |  |  |
| Rv1945 | | 0.0863 | | 0.0270 | |  |  |  |
| Rv2631 | | 0.1332 | | 0.0270 | |  |  |  |
| Rv3615 | | 0.1239 | | 0.0270 | |  |  |  |
| Rv3840 | | 0.0829 | | 0.0270 | |  |  |  |
| Rv0383 | | 0.1433 | | 0.0270 | |  |  |  |
| Rv0468 | | 0.1522 | | 0.0270 | |  |  |  |
| **HIV-** | | | | | | **HIV+** | | |
| **Mtb**  **antigens** | | **log Fold Change**  **ATB/LTB** | | **P-value** | | **Mtb antigens** | **log Fold Change**  **ATB/LTB** | **P-value** |
| Rv0847 | | 0.1920 | | 0.0270 | |  |  |  |
| Rv2004 | | 0.1005 | | 0.0270 | |  |  |  |
| Rv2028 | | 0.1057 | | 0.0270 | |  |  |  |
| Rv2435-N | | 0.1638 | | 0.0270 | |  |  |  |
| Rv2625 | | 0.1861 | | 0.0270 | |  |  |  |
| Rv2657 | | 0.1136 | | 0.0270 | |  |  |  |
| Rv2982 | | 0.1146 | | 0.0270 | |  |  |  |
| Rv0824 | | 0.1284 | | 0.0280 | |  |  |  |
| Rv3229 | | 0.1138 | | 0.0280 | |  |  |  |
| Rv1115 | | 0.1860 | | 0.0286 | |  |  |  |
| Rv3048 | | 0.0902 | | 0.0286 | |  |  |  |
| TB10.4 | | 0.4848 | | 0.0286 | |  |  |  |
| Rv0767 | | 0.1148 | | 0.0298 | |  |  |  |
| Rv1792 | | 0.2479 | | 0.0298 | |  |  |  |
| Rv2380-N | | 0.1308 | | 0.0298 | |  |  |  |
| Rv3133 | | 0.0993 | | 0.0298 | |  |  |  |
| Rv2558 | | 0.0895 | | 0.0301 | |  |  |  |
| Rv2629 | | 0.1154 | | 0.0301 | |  |  |  |
| Rv3128 | | 0.1109 | | 0.0301 | |  |  |  |
| Rv3427 | | 0.1340 | | 0.0301 | |  |  |  |
| Rv0642 | | 0.1146 | | 0.0308 | |  |  |  |
| Rv1884 | | 0.1155 | | 0.0308 | |  |  |  |
| Rv3846 | | 0.1085 | | 0.0308 | |  |  |  |
| Rv0573 | | 0.0879 | | 0.0320 | |  |  |  |
| Rv1479 | | 0.1390 | | 0.0320 | |  |  |  |
| Rv1874 | | 0.1039 | | 0.0320 | |  |  |  |
| Rv2380m | | 0.1175 | | 0.0320 | |  |  |  |
| Rv2642 | | 0.1010 | | 0.0320 | |  |  |  |
| Rv2658 | | 0.0974 | | 0.0320 | |  |  |  |
| Rv0066 | | 0.1340 | | 0.0323 | |  |  |  |
| Rv0570-C | | 0.1245 | | 0.0323 | |  |  |  |
| Rv1528 | | 0.0704 | | 0.0323 | |  |  |  |
| Rv1735 | | 0.1597 | | 0.0323 | |  |  |  |
| Rv2030 | | 0.0775 | | 0.0323 | |  |  |  |
| Rv2346_Rv2347 | | 0.1465 | | 0.0323 | |  |  |  |
| Rv2383-C | | 0.1173 | | 0.0323 | |  |  |  |
| **HIV-** | | | | | **HIV+** | | | |
| **Mtb antigens** | **log Fold Change**  **ATB/LTB** | | **P-value** | | **Mtb**  **antigens** | | **log Fold Change**  **ATB/LTB** | **P-value** |
| Rv2461 | | 0.1118 | | 0.0323 | |  |  |  |
| Rv2913 | | 0.1089 | | 0.0323 | |  |  |  |
| Rv1806 | | 0.0945 | | 0.0331 | |  |  |  |
| Rv1956 | | 0.0950 | | 0.0331 | |  |  |  |
| Rv2710 | | 0.1011 | | 0.0331 | |  |  |  |
| Rv2941 | | 0.0770 | | 0.0331 | |  |  |  |
| Rv3052 | | 0.1292 | | 0.0331 | |  |  |  |
| Rv3536 | | 0.1894 | | 0.0331 | |  |  |  |
| Rv0501 | | 0.1267 | | 0.0334 | |  |  |  |
| Rv1284 | | 0.1057 | | 0.0334 | |  |  |  |
| Rv1733 | | 0.0829 | | 0.0334 | |  |  |  |
| Rv1996 | | 0.1161 | | 0.0334 | |  |  |  |
| Rv2225 | | 0.1241 | | 0.0334 | |  |  |  |
| Rv2987 | | 0.0989 | | 0.0334 | |  |  |  |
| Rv2324 | | 0.1286 | | 0.0363 | |  |  |  |
| Rv2737-N | | 0.1223 | | 0.0363 | |  |  |  |
| Rv2745 | | 0.0793 | | 0.0363 | |  |  |  |
| Rv0353 | | 0.1513 | | 0.0374 | |  |  |  |
| Rv1009 | | 0.1458 | | 0.0374 | |  |  |  |
| Rv1471 | | 0.0788 | | 0.0374 | |  |  |  |
| Rv2435-C | | 0.1676 | | 0.0393 | |  |  |  |
| Rv3127 | | 0.1058 | | 0.0393 | |  |  |  |
| Rv0570-N | | 0.0815 | | 0.0409 | |  |  |  |
| Rv2465 | | 0.1464 | | 0.0409 | |  |  |  |
| Rv2626 | | 0.1029 | | 0.0409 | |  |  |  |
| Rv3126 | | 0.0816 | | 0.0409 | |  |  |  |
| Rv3129 | | 0.0767 | | 0.0409 | |  |  |  |
| PPD | | 0.1638 | | 0.0431 | |  |  |  |
| Rv1221 | | 0.1650 | | 0.0448 | |  |  |  |
| Rv1737 | | 0.1336 | | 0.0448 | |  |  |  |
| Rv1872 | | 0.0874 | | 0.0448 | |  |  |  |
| Rv0574 | | 0.0924 | | 0.0467 | |  |  |  |
| Rv1909 | | 0.2273 | | 0.0467 | |  |  |  |
| Rv2431 | | 0.1898 | | 0.0467 | |  |  |  |
| Rv2035 | | 0.1007 | | 0.0491 | |  |  |  |

| **IgM** | | | | | |
| --- | --- | --- | --- | --- | --- |
| **HIV-** | | | **HIV+** | | |
| **Mtb antigens** | **log Fold Change**  **ATB/LTB** | **P-value** | **Mtb antigens** | **log Fold Change**  **ATB/LTB** | **P-value** |
| Rv3407 | 0.3407 | 0.0320 | Rv1508 | -0.3998 | 0.0218 |
| Rv0246 | 0.2266 | 0.0409 | Rv3132 | -0.4502 | 0.0218 |
| Rv1909 | 0.2363 | 0.0431 | 85A | -0.3352 | 0.0233 |
| Rv1792 | 0.3168 | 0.0448 | Rv1967 | -0.3085 | 0.0266 |
| Rv3132 | 0.1330 | 0.0448 | Rv3134 | -0.3530 | 0.0266 |
|  |  |  | Rv1506 | -0.0929 | 0.0275 |
|  |  |  | Rv0717 | -0.2540 | 0.0290 |
|  |  |  | Rv0251 | -0.1291 | 0.0303 |
|  |  |  | Rv1846 | -0.1948 | 0.0356 |
|  |  |  | Rv1813 | -0.1343 | 0.0377 |
|  |  |  | Hlp | -0.1705 | 0.0408 |
|  |  |  | Rv3131 | -0.1067 | 0.0408 |
|  |  |  | Rv3353 | -0.2465 | 0.0408 |
|  |  |  | 65K | -0.0547 | 0.0441 |
|  |  |  | Rv1512 | -0.1252 | 0.0441 |
|  |  |  | ICL | -0.1090 | 0.0471 |
|  |  |  | PSTS-3 | -0.1009 | 0.0471 |
|  |  |  | Rv1837 | -0.1291 | 0.0471 |
|  |  |  | Rv2466 | -0.1186 | 0.0471 |

| **FcgR2AR** | | | | | |
| --- | --- | --- | --- | --- | --- |
| **HIV-** | | | **HIV+** | | |
| **Mtb antigens** | **log Fold Change**  **ATB/LTB** | **P-value** | **Mtb**  **antigens** | **log Fold Change**  **ATB/LTB** | **P-value** |
| Rv2034 | -0.1126 | 0.0001 | Rv0079 | 0.3358 | 0.0218 |
| Rv0711-C | -0.2304 | 0.0006 | Rv0080 | 0.2724 | 0.0218 |
| Rv2659 | -0.1530 | 0.0007 | Rv0081 | 0.2418 | 0.0218 |
| Rv3353 | -0.0254 | 0.0011 | Rv0140 | 0.2423 | 0.0218 |
| Rv2661 | -0.1059 | 0.0032 | Rv0331 | 0.2486 | 0.0218 |
| Rv1792 | -0.1066 | 0.0062 | Rv0383 | 0.2666 | 0.0218 |
| 85A | -0.0414 | 0.0072 | Rv0384 | 0.3127 | 0.0218 |
| Rv1038 | -0.1911 | 0.0136 | Rv0423 | 0.2733 | 0.0218 |
| Rv1884 | -0.1741 | 0.0150 | Rv0570-C | 0.2862 | 0.0218 |
| Rv2630 | -0.0787 | 0.0150 | Rv0571 | 0.2646 | 0.0218 |
| Rv1997-N | -0.0948 | 0.0163 | Rv0572 | 0.3267 | 0.0218 |
| Rv2658 | -0.2069 | 0.0185 | Rv0574 | 0.2589 | 0.0218 |
| Rv2435-C | -0.2805 | 0.0194 | Rv0645 | 0.2716 | 0.0218 |
| Rv2662 | -0.0881 | 0.0194 | Rv0767 | 0.2850 | 0.0218 |
| Rv1908-C | -0.1732 | 0.0198 | Rv0839 | 0.2850 | 0.0218 |
| Rv1997-C | -0.1611 | 0.0236 | Rv1073 | 0.2422 | 0.0218 |
| Rv2660 | -0.2113 | 0.0236 | Rv1131 | 0.2360 | 0.0218 |
| Rv0711-N | -0.0971 | 0.0239 | Rv1528 | 0.2670 | 0.0218 |
| Rv0753 | -0.1090 | 0.0239 | Rv1717 | 0.2398 | 0.0218 |
| HSP70 | -0.1043 | 0.0240 | Rv1734 | 0.2560 | 0.0218 |
| Rv0640 | -0.1503 | 0.0240 | Rv1736-N | 0.2655 | 0.0218 |
| Rv2737-N | -0.2355 | 0.0240 | Rv1738 | 0.3179 | 0.0218 |
| LAM | 0.4404 | 0.0249 | Rv1791 | 0.2265 | 0.0218 |
| Rv1955 | -0.1741 | 0.0249 | Rv1872 | 0.2676 | 0.0218 |
| Rv0080 | -0.1302 | 0.0254 | Rv1875 | 0.3127 | 0.0218 |
| Rv0824 | -0.2107 | 0.0254 | Rv1884 | 0.1903 | 0.0218 |
| Rv2225 | -0.2007 | 0.0254 | Rv1945 | 0.2936 | 0.0218 |
| Rv2982 | -0.1783 | 0.0263 | Rv1996 | 0.3194 | 0.0218 |
| Rv2737-C | -0.1656 | 0.0270 | Rv1997-N | 0.1451 | 0.0218 |
| Rv1403 | -0.2024 | 0.0298 | Rv1998 | 0.2321 | 0.0218 |
| Rv2657 | -0.1362 | 0.0298 | Rv2004 | 0.2923 | 0.0218 |
| Rv3462 | -0.1043 | 0.0348 | Rv2005 | 0.3109 | 0.0218 |
| Rv0990 | -0.1368 | 0.0363 | Rv2006 | 0.2937 | 0.0218 |
| Rv0991 | -0.1762 | 0.0374 | Rv2028 | 0.2946 | 0.0218 |
| Rv3133 | 0.0891 | 0.0374 | Rv2245 | 0.2710 | 0.0218 |
| **HIV-** | | | **HIV+** | | |
| **Mtb antigens** | **log Fold Change**  **ATB/LTB** | **P-value** | **Mtb**  **antigens** | **log Fold Change**  **ATB/LTB** | **P-value** |
| Rv0246 | -0.0617 | 0.0467 | Rv2346_Rv2347 | 0.2788 | 0.0218 |
| Rv2988 | -0.1107 | 0.0467 | Rv2382 | 0.3142 | 0.0218 |
| Rv2324 | -0.2319 | 0.0491 | Rv2383-C | 0.2925 | 0.0218 |
| Rv2989 | -0.1587 | 0.0491 | Rv2383-N | 0.2825 | 0.0218 |
|  |  |  | Rv2431 | 0.2866 | 0.0218 |
|  |  |  | Rv2461 | 0.2754 | 0.0218 |
|  |  |  | Rv2465 | 0.3268 | 0.0218 |
|  |  |  | Rv2623 | 0.3295 | 0.0218 |
|  |  |  | Rv2627 | 0.3369 | 0.0218 |
|  |  |  | Rv2628 | 0.3203 | 0.0218 |
|  |  |  | Rv2629 | 0.2815 | 0.0218 |
|  |  |  | Rv2631 | 0.3045 | 0.0218 |
|  |  |  | Rv2657 | 0.2648 | 0.0218 |
|  |  |  | Rv2710 | 0.2868 | 0.0218 |
|  |  |  | Rv2745 | 0.2888 | 0.0218 |
|  |  |  | Rv2873 | 0.2093 | 0.0218 |
|  |  |  | Rv2941 | 0.2699 | 0.0218 |
|  |  |  | Rv2987 | 0.2846 | 0.0218 |
|  |  |  | Rv2989 | 0.2995 | 0.0218 |
|  |  |  | Rv3048 | 0.2710 | 0.0218 |
|  |  |  | Rv3052 | 0.2483 | 0.0218 |
|  |  |  | Rv3128 | 0.2859 | 0.0218 |
|  |  |  | Rv3129 | 0.3473 | 0.0218 |
|  |  |  | Rv3307 | 0.2730 | 0.0218 |
|  |  |  | Rv3406 | 0.2995 | 0.0218 |
|  |  |  | Rv3463 | 0.2418 | 0.0218 |
|  |  |  | Rv3583 | 0.3322 | 0.0218 |
|  |  |  | Rv3840 | 0.3034 | 0.0218 |
|  |  |  | Rv3846 | 0.2623 | 0.0218 |
|  |  |  | Rv3862 | 0.3301 | 0.0218 |
|  |  |  | Rv0573 | 0.2617 | 0.0233 |
|  |  |  | Rv1471 | 0.2441 | 0.0233 |
|  |  |  | Rv2626 | 0.3220 | 0.0233 |
|  |  |  | Rv3126 | 0.3029 | 0.0233 |
|  |  |  | Rv3133 | 0.3295 | 0.0233 |
|  |  |  | Rv3615 | 0.2409 | 0.0233 |
| **HIV-** | | | **HIV+** | | |
| **Mtb antigens** | **log Fold Change**  **ATB/LTB** | **P-value** | **Mtb**  **antigens** | **log Fold Change**  **ATB/LTB** | **P-value** |
|  |  |  | PPD | 0.2033 | 0.0251 |
|  |  |  | Rv0753 | 0.2405 | 0.0251 |
|  |  |  | Rv3865 | 0.1775 | 0.0251 |
|  |  |  | Rv1874 | 0.2845 | 0.0261 |
|  |  |  | Rv1980 | 0.1782 | 0.0261 |
|  |  |  | Rv2030 | 0.3213 | 0.0261 |
|  |  |  | Rv2624 | 0.3347 | 0.0261 |
|  |  |  | Rv3127 | 0.3120 | 0.0261 |
|  |  |  | Rv1792 | 0.2682 | 0.0265 |
|  |  |  | Rv1812 | 0.2398 | 0.0265 |
|  |  |  | Rv2630 | 0.1889 | 0.0265 |
|  |  |  | Rv3054 | 0.3253 | 0.0265 |
|  |  |  | Rv3710 | 0.1632 | 0.0265 |
|  |  |  | Rv0570-N | 0.2642 | 0.0266 |
|  |  |  | Rv1806 | 0.1790 | 0.0266 |
|  |  |  | Rv2003 | 0.2457 | 0.0266 |
|  |  |  | Rv0244 | 0.1841 | 0.0275 |
|  |  |  | Rv0501 | 0.1953 | 0.0275 |
|  |  |  | Rv0711-C | 0.1908 | 0.0275 |
|  |  |  | Rv0990 | 0.1731 | 0.0275 |
|  |  |  | Rv1009 | 0.2238 | 0.0275 |
|  |  |  | Rv1038 | 0.2194 | 0.0275 |
|  |  |  | Rv1130 | 0.3042 | 0.0275 |
|  |  |  | Rv1170 | 0.1776 | 0.0275 |
|  |  |  | Rv1403 | 0.1931 | 0.0275 |
|  |  |  | Rv1908-N | 0.1657 | 0.0275 |
|  |  |  | Rv2034 | 0.1928 | 0.0275 |
|  |  |  | Rv2389 | 0.1648 | 0.0275 |
|  |  |  | Rv2558 | 0.1779 | 0.0275 |
|  |  |  | Rv2659 | 0.1900 | 0.0275 |
|  |  |  | Rv2660 | 0.1912 | 0.0275 |
|  |  |  | Rv3019 | 0.2842 | 0.0275 |
|  |  |  | Rv3229 | 0.1532 | 0.0275 |
|  |  |  | Rv3420 | 0.2041 | 0.0275 |
|  |  |  | Rv3536 | 0.1302 | 0.0275 |
|  |  |  |  |  |  |
| **HIV-** | | | **HIV+** | | |
| **Mtb antigens** | **log Fold Change**  **ATB/LTB** | **P-value** | **Mtb**  **antigens** | **log Fold Change**  **ATB/LTB** | **P-value** |
|  |  |  | Rv3614_Rv3615 | 0.2732 | 0.0275 |
|  |  |  | Rv3616 | 0.1566 | 0.0275 |
|  |  |  | Rv1955 | 0.1706 | 0.0279 |
|  |  |  | Rv1957 | 0.1872 | 0.0279 |
|  |  |  | Rv2380m | 0.2025 | 0.0279 |
|  |  |  | Rv2658 | 0.1698 | 0.0279 |
|  |  |  | Rv2913 | 0.1418 | 0.0279 |
|  |  |  | Rv0066 | 0.1872 | 0.0290 |
|  |  |  | Rv0251 | 0.2716 | 0.0290 |
|  |  |  | Rv0468 | 0.1966 | 0.0290 |
|  |  |  | Rv0470 | 0.1824 | 0.0290 |
|  |  |  | Rv0642 | 0.1988 | 0.0290 |
|  |  |  | Rv1390 | 0.1800 | 0.0290 |
|  |  |  | Rv1479 | 0.1944 | 0.0290 |
|  |  |  | Rv1956 | 0.1611 | 0.0290 |
|  |  |  | Rv1997-C | 0.1859 | 0.0290 |
|  |  |  | Rv2324 | 0.1938 | 0.0290 |
|  |  |  | Rv2380-N | 0.1991 | 0.0290 |
|  |  |  | Rv2435-C | 0.1623 | 0.0290 |
|  |  |  | Rv2982 | 0.1828 | 0.0290 |
|  |  |  | Rv0824 | 0.1931 | 0.0292 |
|  |  |  | Rv0967 | 0.1643 | 0.0292 |
|  |  |  | Rv2225 | 0.1977 | 0.0292 |
|  |  |  | Rv2625 | 0.1536 | 0.0292 |
|  |  |  | Rv2642 | 0.1478 | 0.0292 |
|  |  |  | Rv2737-C | 0.1985 | 0.0292 |
|  |  |  | Rv2737-N | 0.1742 | 0.0292 |
|  |  |  | Rv1284 | 0.1933 | 0.0303 |
|  |  |  | Rv2035 | 0.1562 | 0.0303 |
|  |  |  | Rv3427 | 0.2036 | 0.0303 |
|  |  |  | Rv0640 | 0.1753 | 0.0311 |
|  |  |  | Rv1221 | 0.1615 | 0.0311 |
|  |  |  | Rv0711-N | 0.1053 | 0.0331 |
|  |  |  | Rv2215 | 0.1482 | 0.0331 |
|  |  |  | Rv1908-C | 0.1661 | 0.0356 |
|  |  |  | Rv1733 | 0.2186 | 0.0441 |
|  |  |  | Rv2380-C | 0.2114 | 0.0441 |
|  |  |  | Rv2466 | 0.2388 | 0.0441 |
|  |  |  | Rv3515 | 0.1336 | 0.0441 |

| **FcgR2B** | | | | | | | | | | |
| --- | --- | --- | --- | --- | --- | --- | --- | --- | --- | --- |
| **HIV-** | | | | | | **HIV+** | | | | |
| **Mtb**  **antigens** | | **log Fold Change**  **ATB/LTB** | | **P-value** | | **Mtb**  **antigens** | | **log Fold Change**  **ATB/LTB** | | **P-value** |
| Rv2631 | | 0.1948 | | 0.0185 | | 65K | | 0.0996 | | 0.0218 |
| Rv2710 | | 0.1842 | | 0.0185 | | PSTS-3 | | 0.1347 | | 0.0251 |
| LAM | | 1.1749 | | 0.0194 | | 85B | | 0.1459 | | 0.0261 |
| Rv3019 | | 0.1441 | | 0.0194 | | Rv2346_Rv2347 | | 0.2569 | | 0.0261 |
| Rv3406 | | 0.1641 | | 0.0194 | | Rv0383 | | 0.2564 | | 0.0265 |
| Rv2005 | | 0.1819 | | 0.0248 | | Rv3048 | | 0.2445 | | 0.0265 |
| Rv2629 | | 0.1937 | | 0.0248 | | Rv3846 | | 0.2690 | | 0.0265 |
| Rv0569 | | 0.5867 | | 0.0263 | | Rv1038 | | 0.2356 | | 0.0266 |
| Rv0642 | | 0.1906 | | 0.0263 | | Rv1284 | | 0.2272 | | 0.0266 |
| Rv1945 | | 0.1597 | | 0.0263 | | Rv2461 | | 0.2537 | | 0.0266 |
| Rv2383-N | | 0.1764 | | 0.0263 | | Rv3130 | | 0.1474 | | 0.0266 |
| Rv3846 | | 0.1673 | | 0.0263 | | Rv3583 | | 0.2586 | | 0.0266 |
| Rv2346_Rv2347 | | 0.2590 | | 0.0270 | | Hlp | | 0.1466 | | 0.0275 |
| Rv1528 | | 0.1387 | | 0.0270 | | HSP70 | | 0.2788 | | 0.0275 |
| Rv2004 | | 0.1360 | | 0.0270 | | PPD | | 0.1653 | | 0.0275 |
| Rv2324 | | 0.1553 | | 0.0270 | | Rv0066 | | 0.2256 | | 0.0275 |
| Rv3048 | | 0.1985 | | 0.0270 | | Rv0423 | | 0.2778 | | 0.0275 |
| Rv3654 | | 0.5545 | | 0.0270 | | Rv0468 | | 0.2073 | | 0.0275 |
| Rv1736-N | | 0.2096 | | 0.0286 | | Rv0501 | | 0.2348 | | 0.0275 |
| Rv2028 | | 0.1770 | | 0.0298 | | Rv0640 | | 0.2080 | | 0.0275 |
| Rv0383 | | 0.1773 | | 0.0301 | | Rv0642 | | 0.2201 | | 0.0275 |
| Rv0423 | | 0.1436 | | 0.0301 | | Rv0645 | | 0.2353 | | 0.0275 |
| Rv1284 | | 0.1377 | | 0.0301 | | Rv0824 | | 0.2133 | | 0.0275 |
| Rv2006 | | 0.0997 | | 0.0301 | | Rv1390 | | 0.2330 | | 0.0275 |
| Rv2987 | | 0.1603 | | 0.0301 | | Rv1479 | | 0.2235 | | 0.0275 |
| Rv3420 | | 0.1350 | | 0.0320 | | Rv1506 | | 0.1225 | | 0.0275 |
| Rv2558 | | 0.1652 | | 0.0323 | | Rv1507 | | 0.0910 | | 0.0275 |
| Rv1734 | | 0.1465 | | 0.0331 | | Rv1509 | | 0.0986 | | 0.0275 |
| Rv2941 | | 0.1742 | | 0.0331 | | Rv1511 | | 0.1273 | | 0.0275 |
| Rv3840 | | 0.1368 | | 0.0331 | | Rv1791 | | 0.2183 | | 0.0275 |
| Rv0081 | | 0.1755 | | 0.0334 | | Rv1792 | | 0.2172 | | 0.0275 |
| Rv0501 | | 0.1807 | | 0.0334 | | Rv1872 | | 0.2469 | | 0.0275 |
| Rv0645 | | 0.1821 | | 0.0334 | | Rv2225 | | 0.2409 | | 0.0275 |
| Rv2383-C | | 0.1843 | | 0.0334 | | Rv2245 | | 0.2282 | | 0.0275 |
| groES | | 0.1320 | | 0.0348 | | Rv2324 | | 0.2311 | | 0.0275 |
| **HIV-** | | | | | **HIV+** | | | | | |
| **Mtb antigens** | **log Fold Change**  **ATB/LTB** | | **P-value** | | **Mtb**  **antigens** | | **log Fold Change**  **ATB/LTB** | | **P-value** | |
| Rv0990 | | 0.1420 | | 0.0348 | | Rv2380m | | 0.2206 | | 0.0275 |
| Rv2030 | | 0.0569 | | 0.0348 | | Rv2431 | | 0.2357 | | 0.0275 |
| Rv2461 | | 0.2133 | | 0.0348 | | Rv2435-C | | 0.2160 | | 0.0275 |
| Rv1471 | | 0.1599 | | 0.0363 | | Rv2657 | | 0.2256 | | 0.0275 |
| Rv1507 | | 0.0735 | | 0.0370 | | Rv2710 | | 0.2417 | | 0.0275 |
| Rv3126 | | 0.0880 | | 0.0374 | | Rv2737-C | | 0.2499 | | 0.0275 |
| Rv3133 | | 0.1022 | | 0.0374 | | Rv2737-N | | 0.2164 | | 0.0275 |
| PPD | | 0.2317 | | 0.0393 | | Rv2873 | | 0.1926 | | 0.0275 |
| Rv1791 | | 0.2470 | | 0.0393 | | Rv2941 | | 0.2505 | | 0.0275 |
| Rv2745 | | 0.0788 | | 0.0393 | | Rv2982 | | 0.2106 | | 0.0275 |
| Rv1957 | | 0.1156 | | 0.0409 | | Rv3052 | | 0.2475 | | 0.0275 |
| Rv0767 | | 0.1415 | | 0.0431 | | Rv3134 | | 0.1151 | | 0.0275 |
| Rv0573 | | 0.1242 | | 0.0448 | | Rv3420 | | 0.2369 | | 0.0275 |
| Rv0331 | | 0.2246 | | 0.0467 | | Rv3515 | | 0.1875 | | 0.0275 |
| Rv0839 | | 0.1061 | | 0.0467 | | Rv3614_Rv3615 | | 0.2537 | | 0.0275 |
| Rv1717 | | 0.1676 | | 0.0491 | | 19kD | | 0.1392 | | 0.0279 |
| Rv2389 | | 0.1849 | | 0.0491 | | ICL | | 0.0719 | | 0.0279 |
| Rv3307 | | 0.0901 | | 0.0491 | | Rv0470 | | 0.2131 | | 0.0279 |
| Rv3463 | | 0.1933 | | 0.0491 | | Rv1221 | | 0.2253 | | 0.0279 |
|  | |  | |  | | Rv1516 | | 0.1373 | | 0.0279 |
|  | |  | |  | | Rv1717 | | 0.2682 | | 0.0279 |
|  | |  | |  | | Rv1997-N | | 0.3170 | | 0.0279 |
|  | |  | |  | | Rv2380-C | | 0.2749 | | 0.0279 |
|  | |  | |  | | Rv3536 | | 0.3239 | | 0.0279 |
|  | |  | |  | | Rv3615 | | 0.2536 | | 0.0279 |
|  | |  | |  | | groES | | 0.1876 | | 0.0290 |
|  | |  | |  | | Rv0140 | | 0.2604 | | 0.0290 |
|  | |  | |  | | Rv0246 | | 0.2464 | | 0.0290 |
|  | |  | |  | | Rv0570-C | | 0.3124 | | 0.0290 |
|  | |  | |  | | Rv0711-C | | 0.3509 | | 0.0290 |
|  | |  | |  | | Rv0839 | | 0.3076 | | 0.0290 |
|  | |  | |  | | Rv1073 | | 0.2855 | | 0.0290 |
|  | |  | |  | | Rv1131 | | 0.2711 | | 0.0290 |
|  | |  | |  | | Rv1513 | | 0.1312 | | 0.0290 |
|  | |  | |  | | Rv1874 | | 0.2519 | | 0.0290 |
|  | |  | |  | | Rv1945 | | 0.2703 | | 0.0290 |
| **HIV-** | | | | | **HIV+** | | | | | |
| **Mtb antigens** | **log Fold Change**  **ATB/LTB** | | **P-value** | | **Mtb**  **antigens** | | **log Fold Change**  **ATB/LTB** | | **P-value** | |
|  | |  | |  | | Rv2090 | | 0.2436 | | 0.0290 |
|  | |  | |  | | Rv2380-N | | 0.2200 | | 0.0290 |
|  | |  | |  | | Rv2383-C | | 0.3216 | | 0.0290 |
|  | |  | |  | | Rv2630 | | 0.2644 | | 0.0290 |
|  | |  | |  | | Rv2631 | | 0.3301 | | 0.0290 |
|  | |  | |  | | Rv2642 | | 0.3043 | | 0.0290 |
|  | |  | |  | | Rv3019 | | 0.2392 | | 0.0290 |
|  | |  | |  | | Rv3406 | | 0.3324 | | 0.0290 |
|  | |  | |  | | Rv3427 | | 0.2157 | | 0.0290 |
|  | |  | |  | | Rv3865 | | 0.1686 | | 0.0290 |
|  | |  | |  | | Rv3881 | | 0.1021 | | 0.0290 |
|  | |  | |  | | Rv0081 | | 0.2798 | | 0.0292 |
|  | |  | |  | | Rv0244 | | 0.2970 | | 0.0292 |
|  | |  | |  | | Rv0331 | | 0.3006 | | 0.0292 |
|  | |  | |  | | Rv0571 | | 0.2517 | | 0.0292 |
|  | |  | |  | | Rv0572 | | 0.2699 | | 0.0292 |
|  | |  | |  | | Rv0573 | | 0.2604 | | 0.0292 |
|  | |  | |  | | Rv0574 | | 0.2719 | | 0.0292 |
|  | |  | |  | | Rv0753 | | 0.2664 | | 0.0292 |
|  | |  | |  | | Rv1403 | | 0.2848 | | 0.0292 |
|  | |  | |  | | Rv1471 | | 0.2817 | | 0.0292 |
|  | |  | |  | | Rv1515 | | 0.0978 | | 0.0292 |
|  | |  | |  | | Rv1837 | | 0.1546 | | 0.0292 |
|  | |  | |  | | Rv2028 | | 0.3249 | | 0.0292 |
|  | |  | |  | | Rv2382 | | 0.3023 | | 0.0292 |
|  | |  | |  | | Rv2626 | | 0.2459 | | 0.0292 |
|  | |  | |  | | Rv2628 | | 0.2293 | | 0.0292 |
|  | |  | |  | | Rv2629 | | 0.3222 | | 0.0292 |
|  | |  | |  | | Rv2658 | | 0.3598 | | 0.0292 |
|  | |  | |  | | Rv2661 | | 0.2387 | | 0.0292 |
|  | |  | |  | | Rv2662 | | 0.3070 | | 0.0292 |
|  | |  | |  | | Rv2745 | | 0.3040 | | 0.0292 |
|  | |  | |  | | Rv2987 | | 0.3089 | | 0.0292 |
|  | |  | |  | | Rv3126 | | 0.2411 | | 0.0292 |
|  | |  | |  | | Rv3133 | | 0.2438 | | 0.0292 |
|  | |  | |  | | Rv3840 | | 0.2464 | | 0.0292 |
| **HIV-** | | | | | **HIV+** | | | | | |
| **Mtb antigens** | **log Fold Change**  **ATB/LTB** | | **P-value** | | **Mtb**  **antigens** | | **log Fold Change**  **ATB/LTB** | | **P-value** | |
|  | |  | |  | | TCFP_cfp10 | | 0.1848 | | 0.0292 |
|  | |  | |  | | Rv0080 | | 0.2822 | | 0.0303 |
|  | |  | |  | | Rv0384 | | 0.2769 | | 0.0303 |
|  | |  | |  | | Rv0570-N | | 0.2497 | | 0.0303 |
|  | |  | |  | | Rv1115 | | 0.2827 | | 0.0303 |
|  | |  | |  | | Rv1734 | | 0.2512 | | 0.0303 |
|  | |  | |  | | Rv1736-N | | 0.2668 | | 0.0303 |
|  | |  | |  | | Rv1908-N | | 0.4010 | | 0.0303 |
|  | |  | |  | | Rv1996 | | 0.2310 | | 0.0303 |
|  | |  | |  | | Rv1998 | | 0.2903 | | 0.0303 |
|  | |  | |  | | Rv2004 | | 0.2941 | | 0.0303 |
|  | |  | |  | | Rv2383-N | | 0.2855 | | 0.0303 |
|  | |  | |  | | Rv2623 | | 0.2214 | | 0.0303 |
|  | |  | |  | | Rv2624 | | 0.2357 | | 0.0303 |
|  | |  | |  | | Rv2659 | | 0.3391 | | 0.0303 |
|  | |  | |  | | Rv2989 | | 0.3228 | | 0.0303 |
|  | |  | |  | | Rv3463 | | 0.3077 | | 0.0303 |
|  | |  | |  | | TB10.4 | | 0.1218 | | 0.0303 |
|  | |  | |  | | Rv0767 | | 0.2873 | | 0.0311 |
|  | |  | |  | | Rv0990 | | 0.2809 | | 0.0311 |
|  | |  | |  | | Rv1009 | | 0.2842 | | 0.0311 |
|  | |  | |  | | Rv1733 | | 0.2370 | | 0.0311 |
|  | |  | |  | | Rv1737 | | 0.1882 | | 0.0311 |
|  | |  | |  | | Rv1812 | | 0.2618 | | 0.0311 |
|  | |  | |  | | Rv1875 | | 0.2640 | | 0.0311 |
|  | |  | |  | | Rv1884 | | 0.3020 | | 0.0311 |
|  | |  | |  | | Rv1908-C | | 0.3067 | | 0.0311 |
|  | |  | |  | | Rv1955 | | 0.3860 | | 0.0311 |
|  | |  | |  | | Rv1956 | | 0.3108 | | 0.0311 |
|  | |  | |  | | Rv1997-C | | 0.2963 | | 0.0311 |
|  | |  | |  | | Rv2003 | | 0.2746 | | 0.0311 |
|  | |  | |  | | Rv2005 | | 0.2770 | | 0.0311 |
|  | |  | |  | | Rv2030 | | 0.2563 | | 0.0311 |
|  | |  | |  | | Rv2034 | | 0.3194 | | 0.0311 |
|  | |  | |  | | Rv2913 | | 0.3203 | | 0.0311 |
|  | |  | |  | | Rv2988 | | 0.2997 | | 0.0311 |
| **HIV-** | | | | | **HIV+** | | | | | |
| **Mtb antigens** | **log Fold Change**  **ATB/LTB** | | **P-value** | | **Mtb**  **antigens** | | **log Fold Change**  **ATB/LTB** | | **P-value** | |
|  | |  | |  | | Rv3127 | | 0.2835 | | 0.0311 |
|  | |  | |  | | Rv3128 | | 0.2825 | | 0.0311 |
|  | |  | |  | | Rv3129 | | 0.2880 | | 0.0311 |
|  | |  | |  | | Rv3131 | | 0.1530 | | 0.0311 |
|  | |  | |  | | Rv3229 | | 0.3267 | | 0.0311 |
|  | |  | |  | | Rv3307 | | 0.2369 | | 0.0311 |
|  | |  | |  | | Rv3710 | | 0.3082 | | 0.0311 |
|  | |  | |  | | 16kD | | 0.1354 | | 0.0331 |
|  | |  | |  | | Rv0967 | | 0.2864 | | 0.0331 |
|  | |  | |  | | Rv0991 | | 0.2844 | | 0.0331 |
|  | |  | |  | | Rv1170 | | 0.3573 | | 0.0331 |
|  | |  | |  | | Rv1512 | | 0.1059 | | 0.0331 |
|  | |  | |  | | Rv1528 | | 0.2862 | | 0.0331 |
|  | |  | |  | | Rv2006 | | 0.2992 | | 0.0331 |
|  | |  | |  | | Rv2035 | | 0.3249 | | 0.0331 |
|  | |  | |  | | Rv2465 | | 0.2380 | | 0.0331 |
|  | |  | |  | | Rv2558 | | 0.2922 | | 0.0331 |
|  | |  | |  | | Rv2660 | | 0.3227 | | 0.0331 |
|  | |  | |  | | Rv0847 | | 0.2929 | | 0.0356 |
|  | |  | |  | | Rv1806 | | 0.3172 | | 0.0356 |
|  | |  | |  | | Rv1957 | | 0.2849 | | 0.0356 |
|  | |  | |  | | Rv2215 | | 0.1433 | | 0.0356 |
|  | |  | |  | | Rv2389 | | 0.2871 | | 0.0356 |
|  | |  | |  | | Rv2627 | | 0.2414 | | 0.0356 |
|  | |  | |  | | Rv2643 | | 0.2529 | | 0.0356 |
|  | |  | |  | | Rv3054 | | 0.2945 | | 0.0356 |
|  | |  | |  | | Rv3862 | | 0.2545 | | 0.0356 |
|  | |  | |  | | Rv0079 | | 0.2245 | | 0.0377 |
|  | |  | |  | | Rv0251 | | 0.1992 | | 0.0377 |
|  | |  | |  | | Rv1130 | | 0.2161 | | 0.0377 |
|  | |  | |  | | Rv1738 | | 0.2222 | | 0.0377 |
|  | |  | |  | | Rv2466 | | 0.2500 | | 0.0377 |
|  | |  | |  | | Rv2625 | | 0.3337 | | 0.0377 |
|  | |  | |  | | Rv1736-C | | 0.1842 | | 0.0408 |
|  | |  | |  | | Rv2435-N | | 0.1715 | | 0.0408 |
|  | |  | |  | | Rv1735 | | 0.2472 | | 0.0441 |
|  | |  | |  | | Rv2032 | | 0.0890 | | 0.0441 |
|  | |  | |  | | Rv0867 | | 0.1382 | | 0.0471 |
|  | |  | |  | | Rv1980 | | 0.1670 | | 0.0471 |
|  | |  | |  | | Rv2450 | | 0.0644 | | 0.0471 |

| **FcgR3AV** | | | | | |
| --- | --- | --- | --- | --- | --- |
| **HIV-** | | | **HIV+** | | |
| **Mtb antigens** | **log Fold Change**  **ATB/LTB** | **P-value** | **Mtb**  **antigens** | **log Fold Change**  **ATB/LTB** | **P-value** |
| LAM | 0.5017 | 0.0225 | Rv2431 | 0.2046 | 0.0218 |
| Rv0423 | 0.2100 | 0.0249 | Rv2630 | 0.2265 | 0.0233 |
| PPD | 0.2659 | 0.0254 | Hlp | 0.1218 | 0.0261 |
| Rv3406 | 0.2240 | 0.0270 | Rv3536 | 0.2005 | 0.0261 |
| Rv2004 | 0.1682 | 0.0286 | Rv3583 | 0.2576 | 0.0261 |
| Rv2631 | 0.1858 | 0.0286 | PPD | 0.1558 | 0.0265 |
| Rv0990 | 0.1778 | 0.0308 | Rv0079 | 0.2611 | 0.0265 |
| Rv2007 | 0.2423 | 0.0308 | Rv1874 | 0.2676 | 0.0265 |
| Rv3840 | 0.1822 | 0.0320 | Rv1875 | 0.2484 | 0.0265 |
| Rv0839 | 0.1516 | 0.0323 | Rv1996 | 0.2393 | 0.0265 |
| Rv1528 | 0.1645 | 0.0323 | Rv1997-N | 0.2496 | 0.0265 |
| Rv2461 | 0.2022 | 0.0323 | PSTS-3 | 0.0931 | 0.0266 |
| Rv2629 | 0.2287 | 0.0323 | Rv2461 | 0.2051 | 0.0266 |
| Rv0081 | 0.1472 | 0.0331 | Rv2626 | 0.2616 | 0.0266 |
| Rv2028 | 0.2069 | 0.0331 | Rv2710 | 0.2170 | 0.0266 |
| Rv2383-N | 0.1948 | 0.0334 | 38 kD | 0.0659 | 0.0275 |
| Rv1945 | 0.1241 | 0.0348 | 85B | 0.0719 | 0.0275 |
| Rv0767 | 0.1559 | 0.0363 | Rv0066 | 0.1578 | 0.0275 |
| Rv2987 | 0.1726 | 0.0363 | Rv0081 | 0.2370 | 0.0275 |
| Rv0645 | 0.1473 | 0.0374 | Rv0331 | 0.2470 | 0.0275 |
| Rv2710 | 0.2053 | 0.0374 | Rv0383 | 0.1977 | 0.0275 |
| Rv2745 | 0.1537 | 0.0374 | Rv0423 | 0.2115 | 0.0275 |
| Rv0642 | 0.1759 | 0.0393 | Rv0468 | 0.1774 | 0.0275 |
| Rv1284 | 0.1869 | 0.0409 | Rv0470 | 0.1814 | 0.0275 |
| Rv0383 | 0.1919 | 0.0431 | Rv0501 | 0.1605 | 0.0275 |
| Rv0570-C | 0.1518 | 0.0431 | Rv0570-N | 0.2627 | 0.0275 |
| Rv3048 | 0.1631 | 0.0448 | Rv0571 | 0.2573 | 0.0275 |
| Rv2941 | 0.1983 | 0.0467 | Rv0572 | 0.2260 | 0.0275 |
| Rv3052 | 0.1815 | 0.0467 | Rv0573 | 0.2553 | 0.0275 |
| Rv3654 | 0.6155 | 0.0491 | Rv0640 | 0.1616 | 0.0275 |
| Rv2324 | 0.2120 | 0.0498 | Rv0642 | 0.1628 | 0.0275 |
|  |  |  | Rv0645 | 0.2070 | 0.0275 |
|  |  |  | Rv0753 | 0.2442 | 0.0275 |
|  |  |  | Rv0824 | 0.1634 | 0.0275 |
|  |  |  | Rv1038 | 0.1902 | 0.0275 |
| **HIV-** | | | **HIV+** | | |
| **Mtb antigens** | **log Fold Change**  **ATB/LTB** | **P-value** | **Mtb**  **antigens** | **log Fold Change**  **ATB/LTB** | **P-value** |
|  |  |  | Rv1130 | 0.2292 | 0.0275 |
|  |  |  | Rv1131 | 0.2285 | 0.0275 |
|  |  |  | Rv1390 | 0.1598 | 0.0275 |
|  |  |  | Rv1479 | 0.1683 | 0.0275 |
|  |  |  | Rv1509 | 0.0772 | 0.0275 |
|  |  |  | Rv1512 | 0.0716 | 0.0275 |
|  |  |  | Rv1717 | 0.2401 | 0.0275 |
|  |  |  | Rv1734 | 0.2311 | 0.0275 |
|  |  |  | Rv1736-N | 0.2639 | 0.0275 |
|  |  |  | Rv1738 | 0.2671 | 0.0275 |
|  |  |  | Rv1791 | 0.1884 | 0.0275 |
|  |  |  | Rv1792 | 0.1992 | 0.0275 |
|  |  |  | Rv1812 | 0.2159 | 0.0275 |
|  |  |  | Rv1872 | 0.2088 | 0.0275 |
|  |  |  | Rv1998 | 0.2229 | 0.0275 |
|  |  |  | Rv2005 | 0.2141 | 0.0275 |
|  |  |  | Rv2006 | 0.2289 | 0.0275 |
|  |  |  | Rv2028 | 0.2309 | 0.0275 |
|  |  |  | Rv2030 | 0.2650 | 0.0275 |
|  |  |  | Rv2032 | 0.1414 | 0.0275 |
|  |  |  | Rv2225 | 0.1677 | 0.0275 |
|  |  |  | Rv2245 | 0.1959 | 0.0275 |
|  |  |  | Rv2324 | 0.1653 | 0.0275 |
|  |  |  | Rv2346_Rv2347 | 0.2009 | 0.0275 |
|  |  |  | Rv2380-C | 0.2194 | 0.0275 |
|  |  |  | Rv2382 | 0.2672 | 0.0275 |
|  |  |  | Rv2383-C | 0.2238 | 0.0275 |
|  |  |  | Rv2465 | 0.2572 | 0.0275 |
|  |  |  | Rv2624 | 0.2081 | 0.0275 |
|  |  |  | Rv2627 | 0.2353 | 0.0275 |
|  |  |  | Rv2628 | 0.2175 | 0.0275 |
|  |  |  | Rv2657 | 0.2061 | 0.0275 |
|  |  |  | Rv2660 | 0.2402 | 0.0275 |
|  |  |  | Rv2941 | 0.2166 | 0.0275 |
|  |  |  | Rv3019 | 0.2371 | 0.0275 |
|  |  |  | Rv3048 | 0.2074 | 0.0275 |
| **HIV-** | | | **HIV+** | | |
| **Mtb antigens** | **log Fold Change**  **ATB/LTB** | **P-value** | **Mtb**  **antigens** | **log Fold Change**  **ATB/LTB** | **P-value** |
|  |  |  | Rv3052 | 0.1922 | 0.0275 |
|  |  |  | Rv3126 | 0.2333 | 0.0275 |
|  |  |  | Rv3127 | 0.2577 | 0.0275 |
|  |  |  | Rv3129 | 0.2329 | 0.0275 |
|  |  |  | Rv3130 | 0.0627 | 0.0275 |
|  |  |  | Rv3307 | 0.2458 | 0.0275 |
|  |  |  | Rv3463 | 0.2310 | 0.0275 |
|  |  |  | Rv3615 | 0.1920 | 0.0275 |
|  |  |  | Rv3840 | 0.2283 | 0.0275 |
|  |  |  | Rv3846 | 0.2087 | 0.0275 |
|  |  |  | Rv3862 | 0.2312 | 0.0275 |
|  |  |  | Rv3865 | 0.1450 | 0.0275 |
|  |  |  | Rv0574 | 0.2467 | 0.0279 |
|  |  |  | Rv1284 | 0.1741 | 0.0279 |
|  |  |  | Rv1507 | 0.0544 | 0.0279 |
|  |  |  | Rv1511 | 0.0569 | 0.0279 |
|  |  |  | Rv1997-C | 0.2875 | 0.0279 |
|  |  |  | Rv2380-N | 0.1569 | 0.0279 |
|  |  |  | Rv2380m | 0.1757 | 0.0279 |
|  |  |  | Rv2623 | 0.2283 | 0.0279 |
|  |  |  | Rv2658 | 0.1982 | 0.0279 |
|  |  |  | Rv2737-N | 0.1526 | 0.0279 |
|  |  |  | Rv2987 | 0.2026 | 0.0279 |
|  |  |  | Rv2989 | 0.2424 | 0.0279 |
|  |  |  | Rv3054 | 0.3034 | 0.0279 |
|  |  |  | Rv3420 | 0.1640 | 0.0279 |
|  |  |  | Rv3427 | 0.1580 | 0.0279 |
|  |  |  | groES | 0.1800 | 0.0290 |
|  |  |  | Rv0080 | 0.2396 | 0.0290 |
|  |  |  | Rv0140 | 0.2419 | 0.0290 |
|  |  |  | Rv0570-C | 0.2147 | 0.0290 |
|  |  |  | Rv0767 | 0.2092 | 0.0290 |
|  |  |  | Rv0839 | 0.2426 | 0.0290 |
|  |  |  | Rv1471 | 0.2268 | 0.0290 |
|  |  |  | Rv1516 | 0.0740 | 0.0290 |
|  |  |  | Rv1528 | 0.2134 | 0.0290 |
| **HIV-** | | | **HIV+** | | |
| **Mtb antigens** | **log Fold Change**  **ATB/LTB** | **P-value** | **Mtb**  **antigens** | **log Fold Change**  **ATB/LTB** | **P-value** |
|  |  |  | Rv1945 | 0.1949 | 0.0290 |
|  |  |  | Rv1955 | 0.2113 | 0.0290 |
|  |  |  | Rv1980 | 0.1214 | 0.0290 |
|  |  |  | Rv2003 | 0.2588 | 0.0290 |
|  |  |  | Rv2004 | 0.2281 | 0.0290 |
|  |  |  | Rv2034 | 0.2231 | 0.0290 |
|  |  |  | Rv2383-N | 0.2092 | 0.0290 |
|  |  |  | Rv2435-C | 0.1475 | 0.0290 |
|  |  |  | Rv2629 | 0.2158 | 0.0290 |
|  |  |  | Rv2737-C | 0.1600 | 0.0290 |
|  |  |  | Rv2873 | 0.1535 | 0.0290 |
|  |  |  | Rv2982 | 0.1666 | 0.0290 |
|  |  |  | Rv3128 | 0.2185 | 0.0290 |
|  |  |  | Rv3131 | 0.0841 | 0.0290 |
|  |  |  | Rv3133 | 0.2219 | 0.0290 |
|  |  |  | Rv3406 | 0.2284 | 0.0290 |
|  |  |  | Rv3614_Rv3615 | 0.1805 | 0.0290 |
|  |  |  | Rv0251 | 0.1416 | 0.0292 |
|  |  |  | Rv1073 | 0.2177 | 0.0292 |
|  |  |  | Rv1221 | 0.1297 | 0.0292 |
|  |  |  | Rv1403 | 0.1893 | 0.0292 |
|  |  |  | Rv2659 | 0.2113 | 0.0292 |
|  |  |  | Rv3229 | 0.1943 | 0.0292 |
|  |  |  | 16kD | 0.0491 | 0.0303 |
|  |  |  | HSP70 | 0.1942 | 0.0303 |
|  |  |  | ICL | 0.0661 | 0.0303 |
|  |  |  | Rv1806 | 0.2124 | 0.0303 |
|  |  |  | Rv1884 | 0.1901 | 0.0303 |
|  |  |  | Rv1908-C | 0.2059 | 0.0303 |
|  |  |  | 19kD | 0.0851 | 0.0311 |
|  |  |  | Rv1956 | 0.1952 | 0.0311 |
|  |  |  | Rv1957 | 0.1670 | 0.0311 |
|  |  |  | Rv2558 | 0.1948 | 0.0311 |
|  |  |  | Rv2631 | 0.2052 | 0.0311 |
|  |  |  | Rv2642 | 0.1827 | 0.0311 |
|  |  |  | Rv2913 | 0.2026 | 0.0311 |
| **HIV-** | | | **HIV+** | | |
| **Mtb antigens** | **log Fold Change**  **ATB/LTB** | **P-value** | **Mtb**  **antigens** | **log Fold Change**  **ATB/LTB** | **P-value** |
|  |  |  | Rv3710 | 0.1871 | 0.0311 |
|  |  |  | TB10.4 | 0.1015 | 0.0311 |
|  |  |  | Rv0990 | 0.1754 | 0.0331 |
|  |  |  | Rv1170 | 0.1644 | 0.0331 |
|  |  |  | Rv1513 | 0.0205 | 0.0331 |
|  |  |  | Rv1837 | 0.1032 | 0.0331 |
|  |  |  | Rv0711-C | 0.1688 | 0.0356 |
|  |  |  | Rv2466 | 0.2222 | 0.0356 |
|  |  |  | Rv0244 | 0.1721 | 0.0377 |
|  |  |  | Rv2035 | 0.1684 | 0.0377 |
|  |  |  | Rv2090 | 0.2061 | 0.0377 |
|  |  |  | Rv2435-N | 0.1156 | 0.0377 |
|  |  |  | Rv0384 | 0.2080 | 0.0408 |
|  |  |  | Rv1506 | 0.0269 | 0.0408 |
|  |  |  | Rv1908-N | 0.1966 | 0.0408 |
|  |  |  | Rv2988 | 0.1716 | 0.0408 |
|  |  |  | Rv3515 | 0.1373 | 0.0408 |
|  |  |  | ESAT6_CFP10 | 0.1081 | 0.0408 |
|  |  |  | Rv2745 | 0.2227 | 0.0441 |

| **FcgR3B** | | | | | | | | | | |
| --- | --- | --- | --- | --- | --- | --- | --- | --- | --- | --- |
| **HIV-** | | | | | | **HIV+** | | | | |
| **Mtb**  **antigens** | | **log Fold Change**  **ATB/LTB** | | **P-value** | | **Mtb**  **antigens** | | **log Fold Change**  **ATB/LTB** | | **P-value** |
| groES | | 0.4596 | | 0.0032 | | PPD | | 0.2282 | | 0.0218 |
| Rv2461 | | 0.1291 | | 0.0047 | | Rv0383 | | 0.2511 | | 0.0218 |
| Rv0423 | | 0.0807 | | 0.0150 | | Rv0423 | | 0.2470 | | 0.0218 |
| Rv3406 | | 0.1085 | | 0.0163 | | Rv0645 | | 0.2367 | | 0.0218 |
| Rv3840 | | 0.0771 | | 0.0174 | | Rv1791 | | 0.2304 | | 0.0218 |
| Rv0470 | | 0.1184 | | 0.0185 | | Rv1872 | | 0.2511 | | 0.0218 |
| LAM | | 1.4542 | | 0.0185 | | Rv2245 | | 0.2411 | | 0.0218 |
| Rv1284 | | 0.0773 | | 0.0185 | | Rv2346_Rv2347 | | 0.2770 | | 0.0218 |
| Rv2034 | | 0.1148 | | 0.0185 | | Rv2431 | | 0.2716 | | 0.0218 |
| Rv3846 | | 0.0823 | | 0.0185 | | Rv2461 | | 0.2714 | | 0.0218 |
| Rv2225 | | 0.0920 | | 0.0194 | | Rv2657 | | 0.2685 | | 0.0218 |
| Rv0645 | | 0.1144 | | 0.0225 | | Rv2710 | | 0.2812 | | 0.0218 |
| Rv2383-N | | 0.0962 | | 0.0239 | | Rv2941 | | 0.2625 | | 0.0218 |
| Rv0990 | | 0.0703 | | 0.0240 | | Rv3048 | | 0.2633 | | 0.0218 |
| Rv0383 | | 0.0865 | | 0.0249 | | Rv3052 | | 0.2673 | | 0.0218 |
| Rv3019 | | 0.0627 | | 0.0249 | | Rv3583 | | 0.2934 | | 0.0218 |
| PPD | | 0.3159 | | 0.0254 | | Rv3615 | | 0.2371 | | 0.0218 |
| Rv0501 | | 0.0923 | | 0.0263 | | Rv3846 | | 0.2665 | | 0.0218 |
| PSTS-3 | | 0.0374 | | 0.0270 | | Rv3865 | | 0.1836 | | 0.0218 |
| Rv0569 | | 0.3486 | | 0.0270 | | Rv1792 | | 0.2472 | | 0.0251 |
| Rv1736-N | | 0.0808 | | 0.0280 | | Rv3536 | | 0.2228 | | 0.0251 |
| Rv2346_Rv2347 | | 0.1018 | | 0.0280 | | Rv3614_Rv3615 | | 0.2726 | | 0.0251 |
| Rv2662 | | 0.1055 | | 0.0280 | | Rv2873 | | 0.2095 | | 0.0261 |
| Rv1791 | | 0.1112 | | 0.0298 | | Rv1980 | | 0.1719 | | 0.0265 |
| Rv3614_Rv3615 | | 0.1100 | | 0.0298 | | Rv0470 | | 0.2039 | | 0.0266 |
| Rv2245 | | 0.1250 | | 0.0301 | | Rv1038 | | 0.2154 | | 0.0266 |
| Rv3420 | | 0.0610 | | 0.0308 | | Rv1390 | | 0.2032 | | 0.0266 |
| Rv2631 | | 0.0665 | | 0.0334 | | Rv1875 | | 0.2326 | | 0.0266 |
| Rv2657 | | 0.1004 | | 0.0334 | | Rv2982 | | 0.2288 | | 0.0266 |
| Rv2745 | | 0.0506 | | 0.0334 | | Rv0066 | | 0.1992 | | 0.0275 |
| Rv3048 | | 0.0886 | | 0.0334 | | Rv0468 | | 0.2118 | | 0.0275 |
| 85B | | 0.0403 | | 0.0348 | | Rv0501 | | 0.2199 | | 0.0275 |
| Rv2558 | | 0.0718 | | 0.0348 | | Rv0571 | | 0.1996 | | 0.0275 |
| Rv2005 | | 0.0526 | | 0.0363 | | Rv0640 | | 0.1729 | | 0.0275 |
| Rv3052 | | 0.0815 | | 0.0363 | | Rv0642 | | 0.2121 | | 0.0275 |
| **HIV-** | | | | | **HIV+** | | | | | |
| **Mtb antigens** | **log Fold Change**  **ATB/LTB** | | **P-value** | | **Mtb**  **antigens** | | **log Fold Change**  **ATB/LTB** | | **P-value** | |
| Rv3427 | | 0.0699 | | 0.0363 | | Rv0711-C | | 0.2482 | | 0.0275 |
| Rv0081 | | 0.0604 | | 0.0374 | | Rv0824 | | 0.2147 | | 0.0275 |
| Rv2659 | | 0.0882 | | 0.0374 | | Rv1221 | | 0.1848 | | 0.0275 |
| Rv2629 | | 0.0389 | | 0.0397 | | Rv1284 | | 0.2157 | | 0.0275 |
| Rv1511 | | 0.0686 | | 0.0409 | | Rv1403 | | 0.2227 | | 0.0275 |
| Rv3865 | | 0.1849 | | 0.0409 | | Rv1479 | | 0.2075 | | 0.0275 |
| 38 kD | | 0.0509 | | 0.0431 | | Rv1806 | | 0.2167 | | 0.0275 |
| Rv1507 | | 0.0392 | | 0.0445 | | Rv1908-N | | 0.2101 | | 0.0275 |
| Rv3616 | | 0.1694 | | 0.0445 | | Rv1955 | | 0.2136 | | 0.0275 |
| Rv0331 | | 0.0939 | | 0.0448 | | Rv1997-C | | 0.2286 | | 0.0275 |
| Rv1471 | | 0.1178 | | 0.0448 | | Rv1997-N | | 0.1415 | | 0.0275 |
| Rv0839 | | 0.0782 | | 0.0467 | | Rv2215 | | 0.1464 | | 0.0275 |
| Rv1515 | | 0.0681 | | 0.0467 | | Rv2225 | | 0.2071 | | 0.0275 |
| Rv2710 | | 0.0650 | | 0.0467 | | Rv2324 | | 0.2292 | | 0.0275 |
| Rv1957 | | 0.0655 | | 0.0491 | | Rv2380m | | 0.2251 | | 0.0275 |
|  | |  | |  | | Rv2435-C | | 0.2145 | | 0.0275 |
|  | |  | |  | | Rv2558 | | 0.2189 | | 0.0275 |
|  | |  | |  | | Rv2624 | | 0.2537 | | 0.0275 |
|  | |  | |  | | Rv2642 | | 0.1958 | | 0.0275 |
|  | |  | |  | | Rv2658 | | 0.1984 | | 0.0275 |
|  | |  | |  | | Rv2659 | | 0.1914 | | 0.0275 |
|  | |  | |  | | Rv2660 | | 0.2130 | | 0.0275 |
|  | |  | |  | | Rv2737-C | | 0.2270 | | 0.0275 |
|  | |  | |  | | Rv2737-N | | 0.2051 | | 0.0275 |
|  | |  | |  | | Rv2913 | | 0.2102 | | 0.0275 |
|  | |  | |  | | Rv3126 | | 0.2316 | | 0.0275 |
|  | |  | |  | | Rv3129 | | 0.2321 | | 0.0275 |
|  | |  | |  | | Rv3229 | | 0.2219 | | 0.0275 |
|  | |  | |  | | Rv3420 | | 0.2085 | | 0.0275 |
|  | |  | |  | | Rv3427 | | 0.2222 | | 0.0275 |
|  | |  | |  | | Rv3710 | | 0.2222 | | 0.0275 |
|  | |  | |  | | Rv0080 | | 0.2682 | | 0.0279 |
|  | |  | |  | | Rv1956 | | 0.2219 | | 0.0279 |
|  | |  | |  | | Rv1957 | | 0.1874 | | 0.0279 |
|  | |  | |  | | Rv2380-N | | 0.2094 | | 0.0279 |
|  | |  | |  | | Rv3127 | | 0.2449 | | 0.0279 |
| **HIV-** | | | | | **HIV+** | | | | | |
| **Mtb antigens** | **log Fold Change**  **ATB/LTB** | | **P-value** | | **Mtb**  **antigens** | | **log Fold Change**  **ATB/LTB** | | **P-value** | |
|  | |  | |  | | Rv3307 | | 0.2141 | | 0.0279 |
|  | |  | |  | | Rv3515 | | 0.2027 | | 0.0279 |
|  | |  | |  | | Rv0331 | | 0.1841 | | 0.0290 |
|  | |  | |  | | Rv0572 | | 0.2272 | | 0.0290 |
|  | |  | |  | | Rv0574 | | 0.2123 | | 0.0290 |
|  | |  | |  | | Rv0967 | | 0.1940 | | 0.0290 |
|  | |  | |  | | Rv0990 | | 0.1980 | | 0.0290 |
|  | |  | |  | | Rv1073 | | 0.2168 | | 0.0290 |
|  | |  | |  | | Rv1736-N | | 0.2100 | | 0.0290 |
|  | |  | |  | | Rv1738 | | 0.2236 | | 0.0290 |
|  | |  | |  | | Rv2030 | | 0.2354 | | 0.0290 |
|  | |  | |  | | Rv2628 | | 0.2581 | | 0.0290 |
|  | |  | |  | | Rv2630 | | 0.1669 | | 0.0290 |
|  | |  | |  | | Rv3133 | | 0.2493 | | 0.0290 |
|  | |  | |  | | Rv3616 | | 0.1695 | | 0.0290 |
|  | |  | |  | | Rv1506 | | 0.1683 | | 0.0292 |
|  | |  | |  | | Rv1509 | | 0.1472 | | 0.0292 |
|  | |  | |  | | Rv1516 | | 0.1525 | | 0.0292 |
|  | |  | |  | | Rv1812 | | 0.2085 | | 0.0292 |
|  | |  | |  | | Rv1996 | | 0.2341 | | 0.0292 |
|  | |  | |  | | Rv2034 | | 0.2234 | | 0.0292 |
|  | |  | |  | | Rv2035 | | 0.1987 | | 0.0292 |
|  | |  | |  | | Rv2380-C | | 0.2615 | | 0.0292 |
|  | |  | |  | | Rv2627 | | 0.2413 | | 0.0292 |
|  | |  | |  | | Rv3130 | | 0.1406 | | 0.0292 |
|  | |  | |  | | Rv0079 | | 0.2189 | | 0.0303 |
|  | |  | |  | | Rv0244 | | 0.2089 | | 0.0303 |
|  | |  | |  | | Rv1130 | | 0.2439 | | 0.0303 |
|  | |  | |  | | Rv1874 | | 0.2115 | | 0.0303 |
|  | |  | |  | | Rv2626 | | 0.2325 | | 0.0303 |
|  | |  | |  | | Rv3019 | | 0.2237 | | 0.0303 |
|  | |  | |  | | Rv0140 | | 0.2035 | | 0.0311 |
|  | |  | |  | | Rv0384 | | 0.2406 | | 0.0311 |
|  | |  | |  | | Rv1170 | | 0.1634 | | 0.0311 |
|  | |  | |  | | Rv1513 | | 0.1090 | | 0.0311 |
|  | |  | |  | | Rv1717 | | 0.2074 | | 0.0311 |
| **HIV-** | | | | | **HIV+** | | | | | |
| **Mtb antigens** | **log Fold Change**  **ATB/LTB** | | **P-value** | | **Mtb**  **antigens** | | **log Fold Change**  **ATB/LTB** | | **P-value** | |
|  | |  | |  | | Rv3463 | | 0.2094 | | 0.0311 |
|  | |  | |  | | Rv0767 | | 0.1778 | | 0.0331 |
|  | |  | |  | | Rv0991 | | 0.2512 | | 0.0331 |
|  | |  | |  | | Rv1734 | | 0.2122 | | 0.0331 |
|  | |  | |  | | Rv1884 | | 0.1661 | | 0.0331 |
|  | |  | |  | | Rv1908-C | | 0.2025 | | 0.0331 |
|  | |  | |  | | Rv2090 | | 0.1980 | | 0.0331 |
|  | |  | |  | | Rv2466 | | 0.2179 | | 0.0331 |
|  | |  | |  | | Rv2623 | | 0.2264 | | 0.0331 |
|  | |  | |  | | Rv2661 | | 0.1979 | | 0.0331 |
|  | |  | |  | | Rv3054 | | 0.2702 | | 0.0331 |
|  | |  | |  | | HSP70 | | 0.2294 | | 0.0356 |
|  | |  | |  | | Rv0081 | | 0.2044 | | 0.0356 |
|  | |  | |  | | Rv0711-N | | 0.1838 | | 0.0356 |
|  | |  | |  | | Rv1131 | | 0.2114 | | 0.0356 |
|  | |  | |  | | Rv1471 | | 0.1744 | | 0.0356 |
|  | |  | |  | | Rv1507 | | 0.1725 | | 0.0356 |
|  | |  | |  | | Rv0251 | | 0.2494 | | 0.0377 |
|  | |  | |  | | Rv0573 | | 0.1993 | | 0.0377 |
|  | |  | |  | | Rv1945 | | 0.1856 | | 0.0377 |
|  | |  | |  | | Rv1998 | | 0.1909 | | 0.0377 |
|  | |  | |  | | Rv2028 | | 0.1946 | | 0.0377 |
|  | |  | |  | | Rv2382 | | 0.2050 | | 0.0377 |
|  | |  | |  | | Rv2383-C | | 0.1801 | | 0.0377 |
|  | |  | |  | | Rv2988 | | 0.1883 | | 0.0377 |
|  | |  | |  | | Rv2989 | | 0.1998 | | 0.0377 |
|  | |  | |  | | Rv3128 | | 0.1798 | | 0.0377 |
|  | |  | |  | | Rv3406 | | 0.1961 | | 0.0377 |
|  | |  | |  | | Rv3840 | | 0.1653 | | 0.0377 |
|  | |  | |  | | Rv3862 | | 0.2421 | | 0.0377 |
|  | |  | |  | | Rv0839 | | 0.1926 | | 0.0408 |
|  | |  | |  | | Rv2465 | | 0.2340 | | 0.0408 |
|  | |  | |  | | Rv2987 | | 0.1646 | | 0.0408 |
|  | |  | |  | | Rv2003 | | 0.1901 | | 0.0441 |
|  | |  | |  | | Rv2383-N | | 0.1853 | | 0.0441 |
|  | |  | |  | | Rv2662 | | 0.2256 | | 0.0441 |
| **HIV-** | | | | | **HIV+** | | | | | |
| **Mtb antigens** | **log Fold Change**  **ATB/LTB** | | **P-value** | | **Mtb**  **antigens** | | **log Fold Change**  **ATB/LTB** | | **P-value** | |
|  | |  | |  | | Rv0570-N | | 0.1944 | | 0.0471 |
|  | |  | |  | | Rv2005 | | 0.1944 | | 0.0471 |
|  | |  | |  | | Rv2006 | | 0.1627 | | 0.0471 |
|  | |  | |  | | Rv2631 | | 0.1790 | | 0.0471 |
|  | |  | |  | | TB10.4 | | 0.1601 | | 0.0471 |
